# Supplementary material for: Comparison of health care resource utilization among preterm and term infants hospitalized with Human Respiratory Syncytial Virus infections: A systematic review and meta-analysis of retrospective cohort studies
Source: PLoS One. 2020 Feb 21;15(2):e0229357. doi: 10.1371/journal.pone.0229357 (PMC7034889; doi:10.1371/journal.pone.0229357)

2.1. Supplemental Figure 1. Funnel plot for publications for preterm and term children admission in intensive care unit.

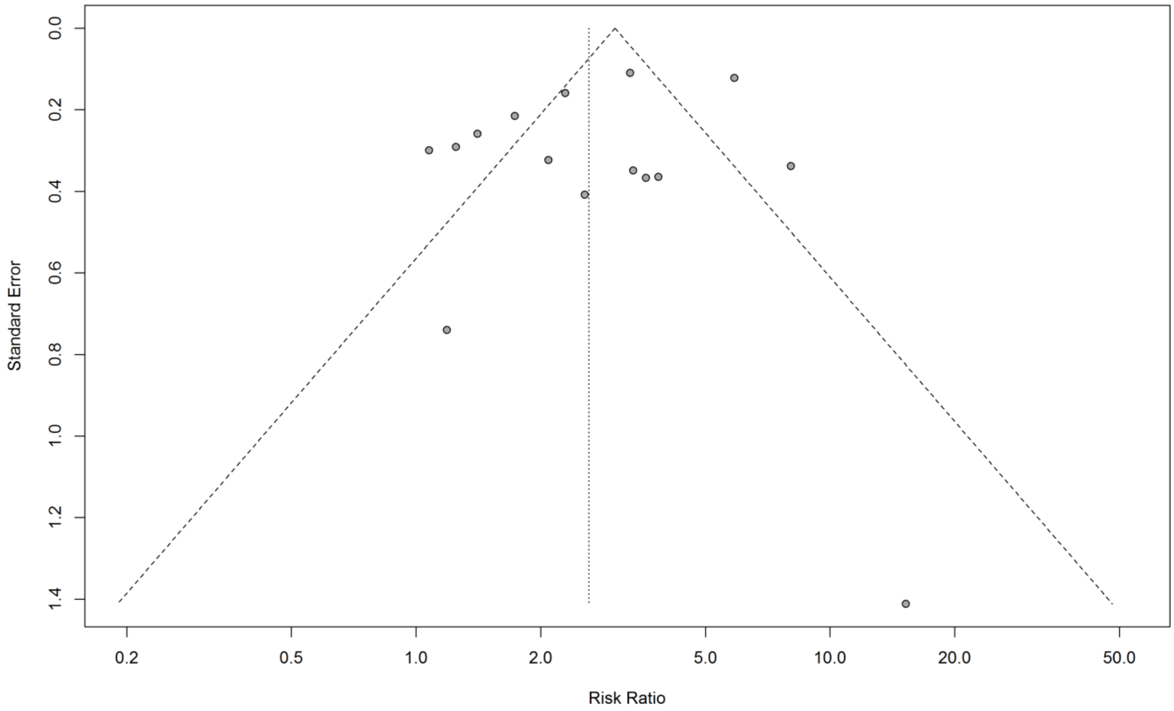

Supplement: S1 Fig — (PDF) [file pone.0229357.s001.pdf]
