# Supplementary material for: Comparison of health care resource utilization among preterm and term infants hospitalized with Human Respiratory Syncytial Virus infections: A systematic review and meta-analysis of retrospective cohort studies
Source: PLoS One. 2020 Feb 21;15(2):e0229357. doi: 10.1371/journal.pone.0229357 (PMC7034889; doi:10.1371/journal.pone.0229357)

2.3. Supplemental Figure 3. Funnel plot for publications for preterm and term children utilization of mechanical ventilation.

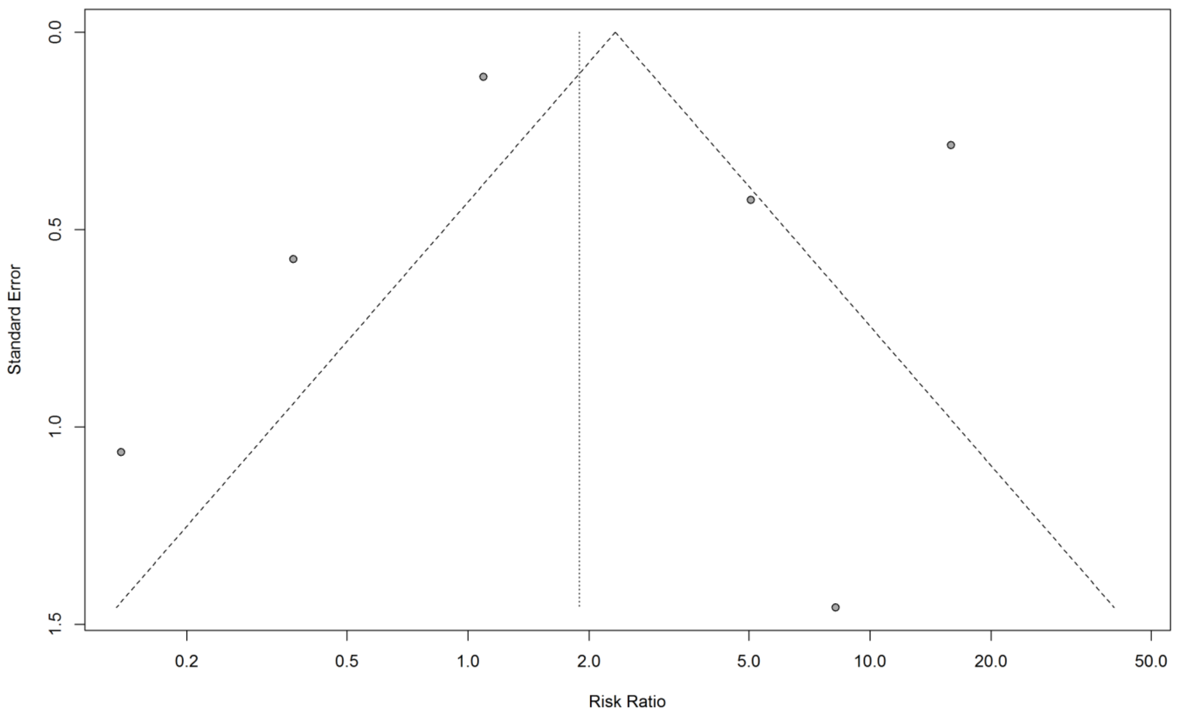

Supplement: S3 Fig — (PDF) [file pone.0229357.s003.pdf]
