# Supplementary material for: Comparison of health care resource utilization among preterm and term infants hospitalized with Human Respiratory Syncytial Virus infections: A systematic review and meta-analysis of retrospective cohort studies
Source: PLoS One. 2020 Feb 21;15(2):e0229357. doi: 10.1371/journal.pone.0229357 (PMC7034889; doi:10.1371/journal.pone.0229357)
Supplement: S2 Table — (PDF) [file pone.0229357.s010.pdf]

1.2. Supplemental Table 2. Search strategy in Embase

No	Query	Results	Date
#1	'hrsv' OR 'rsv' OR 'human respiratory syncytial virus'/exp OR 'human respiratory syncytial virus' OR 'respiratory syncytial virus'/exp OR 'respiratory syncytial virus' OR 'hrsv-a' OR 'hrsv-b'	26.588	22 Jul 2019
#2	preterm* OR prematur* OR gestation* OR term*	3.473.760	22 Jul 2019
#3	hospital* OR inpatient* OR outpatient* OR 'length of stay' OR 'emergency' OR 'icu' OR 'intensive care unit' OR 'intensive care units, neonatal' OR 'hospitalization rate' OR 'rehospitalization' OR 'case fatality rate' OR cfr OR 'mortality'	9.075.253	22 Jul 2019
#4	#1 AND #2 AND #3	2.626	22 Jul 2019
#5	#1 AND #2 AND #3 AND [humans]/lim	2.414	22 Jul 2019
