# Supplementary material for: Comparison of health care resource utilization among preterm and term infants hospitalized with Human Respiratory Syncytial Virus infections: A systematic review and meta-analysis of retrospective cohort studies
Source: PLoS One. 2020 Feb 21;15(2):e0229357. doi: 10.1371/journal.pone.0229357 (PMC7034889; doi:10.1371/journal.pone.0229357)
Supplement: S3 Table — (PDF) [file pone.0229357.s011.pdf]

1.3. Supplemental Table 3. Items for risk of bias assessment

**Newcastle – Ottawa Scale for case cohort studies**	**One star (1)**	**No star (0)**
**Selection**		
1) Representativeness of the HRSV hospitalized preterm cohort	Representative of the population source	Specific population or no description of the population source
2) Selection of the HRSV hospitalized term cohort	From the same population source	From a different population source or no description of the population source
3) Ascertainment of gestational age and HRSV infection	From secure record, structured interview or lab analysis	Self report or no description
4) Demonstration that the use of medical care was not present at the beginning of the study	Yes	No
**Comparability**		
1) Comparability of preterm and term infants for age at inclusion	Yes	No
2) Comparability of preterm and term infants for a second important factor	Yes	No
**Outcome**		
1) Assessment of the use of medical care	Independent blind assessment or record linkage	Self report or no description
2) Was follow-up long enough for the use of medical care	Yes	No
3) Complete follow up of all subjects accounted for 80%	Yes	No or no description
Total score	**9**	**0**

**Interpretation of the two risk of bias tools**<br><br>• 6-9: Low risk of bias<br>• 0-5: High risk of bias		
