# Supplementary material for: Comparison of health care resource utilization among preterm and term infants hospitalized with Human Respiratory Syncytial Virus infections: A systematic review and meta-analysis of retrospective cohort studies
Source: PLoS One. 2020 Feb 21;15(2):e0229357. doi: 10.1371/journal.pone.0229357 (PMC7034889; doi:10.1371/journal.pone.0229357)
Supplement: S4 Table — (PDF) [file pone.0229357.s012.pdf]

1.4. Supplemental Table 4. Main reasons of exclusion of eligible studies

No ID	Author , Year	Title	Reason of exclusion
**1**	Alan, 2016	Outcome of the Respiratory Syncytial Virus related acute lower respiratory tract infection among hospitalized newborns: A prospective multicenter study.	No data on outcomes
**2**	Alverson, 2013	The clinical management of preterm infants with bronchiolitis.	No data on outcomes
**3**	Assandri Dávila, 2005	Neonatal hospitalization through a hospital emergency service in Uruguay.	Term infants not recruted
**4**	Aydiner, 2005	Rehospitalization rates of infants of less than 32 weeks gestation in the first year of life.	No data on outcomes
**5**	Berger, 2009	Prospective population-based study of rsv-related intermediate care and intensive care unit admissions in Switzerland over a 4-year period (2001-2005).	Term infant case definition not appropriate
**6**	Bonillo Perales, 2000	Perinatal history and hospitalization for bronchiolitis. A comparison with the impact-RSV Study Group.	No data on outcomes
**7**	Boyce, 2000	Rates of hospitalization for respiratory syncytial virus infection among children in Medicaid.	Term infants not recruted
**8**	Breuer, 2017	Respiratory Hospitalizations and Rehospitalizations in Infants Born Late Preterm.	No data on outcomes
**9**	Chirikov, 2019	Economic Burden Trajectories in Commercially Insured US Infants with Respiratory Syncytial Virus.	No data on outcomes
**10**	Chu, 2016	Respiratory syncytial virus infection in infants in rural Nepal.	No data on outcomes
**11**	Colin, 2010	The Forgotten Majority - Late preterm infants and their vulnerability to lung disease.	Review
**12**	Cordero, 2018	Impact of respiratory syncytial virus infection and palivizumab on severe acute lower respiratory infection during the first 6 months of life in a cohort of Colombian infants.	No data on outcomes
**13**	El Basha, 2017	Prematurity is a significant predictor of worse outcomes in viral bronchiolitis: A comparative study in infancy.	No data on outcomes
**14**	Fauroux, 2011	Hospitalization for bronchiolitis in very preterm infants without broncho-pulmonary dysplasia.	No data on outcomes
**15**	Fauroux,	Respiratory morbidity of preterm infants of less	No data on

	2014	than 33 weeks gestation without bronchopulmonary dysplasia: A 12-month follow-up of the CASTOR study cohort.	outcomes
**16**	Fergie, 2018	Respiratory syncytial virus hospitalization rates among term and preterm infants before and after changes to the American academy of pediatrics guidance on immunoprophylaxis, 2011-2017.	No data on outcomes
**17**	Fridman, 2016	Three days coming three days staying: the course of respiratory syncytial virus bronchiolitis.	No data on outcomes
**18**	Fryzek, 2011	Trends in chronologic age and infant respiratory syncytial virus hospitalization: An 8-year cohort study.	No data on gestationnal age
**19**	García-Garcia, 2015	Clinical and virological characteristics of early and moderate preterm infants readmitted with viral respiratory infections.	No data on outcomes
**20**	Goldstein, 2018	Respiratory Syncytial Virus Hospitalizations among US. Preterm Infants Compared with Term Infants before and after the 2014 American Academy of Pediatrics Guidance on Immunoprophylaxis: 2012-2016.	Impossible to extract data of interest
**21**	Gooch, 2011	Comparison of risk factors between preterm and term infants hospitalized for severe respiratory syncytial virus in the Russian Federation.	No data on outcomes
**22**	Gouyon, 2013	Hospitalizations for respiratory syncytial virus bronchiolitis in preterm infants at <33 weeks gestation without bronchopulmonary dysplasia: the CASTOR study.	No data on outcomes
**23**	Greenberg, 2019	Incidence of respiratory syncytial virus bronchiolitis in hospitalized infants born at 33–36 weeks of gestational age compared with those born at term: a retrospective cohort study.	No data on outcomes
**24**	Greenough, 2015	Lung function of preterm infants before and after viral infections.	Review
**25**	Gunville, 2010	Scope and impact of early and late preterm infants admitted to the PICU with respiratory illness.	Not Acute Respiratory Infections
**26**	Hall, 2013	Respiratory syncytial virus-associated hospitalizations among children less than 24 months of age.	No data on outcomes
**27**	Homaira, 2016	High burden of RSV hospitalization in very young children: A data linkage study.	No data on outcomes
**28**	Igde, 2016	The investigation of recurrent wheezing frequency in palivizumab applied babies.	No data on outcomes
**29**	Jain, 2015	Age-related effect of viral-induced wheezing in severe prematurity.	No data on outcomes
**30**	Kosma, 2014	Bronchiolitis during the first year after birth in term and preterm infants.	No data on outcomes
**31**	Krilov, 2017	National bronchiolitis hospitalization rates among preterm and full term infants: 2010-2015.	No data on outcomes

**32**	Kristensen, 1998	Epidemiology of respiratory syncytial virus infection requiring hospitalization in East Denmark.	No data on outcomes
**33**	Lanari, 2015	Risk factors for bronchiolitis hospitalization during the first year of life in a multicenter Italian birth cohort	No data on outcomes
**34**	Ledbetter, 2018	Healthcare costs after hospitalization for respiratory syncytial virus or unspecified bronchiolitis in the first year of life.	No data on outcomes
**35**	Lode, 2004	Emergencies in near-term or full-term newborns.	No data on outcomes
**36**	Martín Masot, 2016	Incidence of hospitalization for acute respiratory syncytial virus bronchiolitis in the first year of age in a third level hospital. Have the preterm infants more risk?	No data on outcomes
**37**	McLaurin, 2016	Respiratory syncytial virus hospitalization outcomes and costs of full-term and preterm infants.	No data on outcomes
**38**	Min, 2011	Analysis of palivizumab prophylaxis in patients with acute lower respiratory tract infection caused by respiratory syncytial virus	No data on outcomes
**39**	Murray, 2012	Creating a birth cohort to examine RSV bronchiolitis hospital admission rates among term and preterm infants in england.	No data on outcomes
**40**	Murray, 2014	Risk factors for hospital admission with RSV bronchiolitis in England: A population-based birth cohort study.	No data on outcomes
**41**	Okuonghae, 1992	Nosocomial respiratory syncytial virus infection in a newborn nursery.	No data on outcomes
**42**	Olabarrieta, 2015	Hospital admission due to respiratory viral infections in moderate preterm, late preterm and term infants during their first year of life.	No data on outcomes
**43**	Palmer, 2010	Healthcare costs within a year of respiratory syncytial virus among medicaid infants.	No data on outcomes
**44**	Pancham, 2014	Human metapneumovirus is associated with severe respiratory disease in premature children.	No data on outcomes
**45**	Paramore, 2010	Outpatient RSV lower respiratory infections among high-risk infants and other pediatric populations.	No data on outcomes
**46**	Parikh, 2017	Chronologic Age at Hospitalization for Respiratory Syncytial Virus Among Preterm and Term Infants in the United States.	Review
**47**	Pérez Pérez, 2004	Respiratory morbidity after hospital discharge in premature infants born at ≤ 32 weeks' gestation with bronchopulmonary dysplasia.	No data on outcomes
**48**	Pérez Pérez, 2010	**Bronchopulmonary dysplasia** and prematurity. Short- and long-term respiratory changes.	Review
**49**	Pramana, 2013	Follow up care of the preterm infant.	Review
**50**	Resch, 2002	The impact of respiratory syncytial virus infection: A prospective study in hospitalized infants younger than 2 years.	No data on outcomes

**51**	Resch, 2003	Respiratory syncytial virus in respiratory tract infections in hospitalized children under 2 years.	No data on outcomes
**52**	Resch, 2011	Are late preterm infants as susceptible to RSV infection as full term infants?	Review
**53**	Richard, 2008	The impact of dual viral infection in infants admitted to a pediatric intensive care unit associated with severe bronchiolitis.	No data on outcomes
**54**	Shi, 2011	Association of RSV lower respiratory tract infection and subsequent healthcare use and costs: A Medicaid claims analysis in early-preterm, late-preterm, and full-term infants.	No data on outcomes
**55**	Svensson, 2015	Incidence, risk factors and hospital burden in children under five years of age hospitalised with respiratory syncytial virus infections.	No data on outcomes
**56**	Tammela, 1992	First-year infections after initial hospitalization in low birth weight infants with and without bronchopulmonary dysplasia.	Only low birth weigh and BPD infants included
**57**	Trisorus, 2012	Clinical severity and iPFT compare between subgroups of respiratory syncytial virus in children hospitalized with acute lower respiratory tract infection.	No data on outcomes
**58**	Tsolia, 2013	Direct and indirect burden associated with parents whose infant has been hospitalized for a lower respiratory tract infection (LRTI) in Greece.	No data on outcomes
**59**	Van De Steen, 2012	Respiratory syncytial virus as cause of lower respiratory tract infection in young children in central and Eastern Europe.	Duplicates of Van de Steen, 2016
**60**	Verboon-Maciolek, 2005	Clinical and epidemiologic characteristics of viral infections in a neonatal intensive care unit during a 12-year period.	No data on outcomes
**61**	Willwerth, 2006	Identifying hospitalized infants who have bronchiolitis and are at high risk for apnea.	No data on outcomes
**62**	Winterstein, 2013	Appropriateness of age thresholds for respiratory syncytial virus immunoprophylaxis in moderate-preterm infants: a cohort study.	No data on outcomes
