# Supplementary material for: Comparison of health care resource utilization among preterm and term infants hospitalized with Human Respiratory Syncytial Virus infections: A systematic review and meta-analysis of retrospective cohort studies
Source: PLoS One. 2020 Feb 21;15(2):e0229357. doi: 10.1371/journal.pone.0229357 (PMC7034889; doi:10.1371/journal.pone.0229357)
Supplement: S5 Table — (PDF) [file pone.0229357.s013.pdf]

1.5. Supplemental Table 5. Individual characteristics of included studies

Study ID	Author, year	Age at inclusion	Recruitment period	Country	GA preterm	GA term	Infection	HRSV Prophylaxis	detection assay
1	Assefa, 2011	< 6 months	Unclear/Not reported	USA	< 37 wGA	≥ 37 wGA	First HRSV bronchiolitis	No	Immunofluorescence and cell culture
2	Chi, 2018	< 2 years	2007-2009	Taiwan	< 35 wGA	≥ 37 wGA	HRSV infection	No	International Classification of Diseases code
3	Followell, 2018	Unclear/Not reported	2012-2017	United Kingdom	34-36 wGA	≥ 37 wGA	HRSV bronchiolitis	Unclear/Not reported	Unclear/Not reported
4	Forbes, 2010, ≤32 wGA	≤ 1 year	Jan/2003-Jun/2007	USA	≤ 32 wGA	≥ 37 wGA	HRSV infection	Unclear/Not reported	International Classification of Diseases code
5	Forbes, 2010, 33–36 wGA	≤ 1 year	Jan/2003-Jun/2007	USA	33-36 wGA	≥ 37 wGA	HRSV infection	Unclear/Not reported	International Classification of Diseases code
6	Gijtenbeek, 2015, < 32 wGA	< 5 years	Jan/2002-Dec/2003	Netherlands	< 32 wGA	38-41 wGA	HRSV infection	Yes	RSV antigen detection or cell culture
7	Gijtenbeek, 2015, 32-36 wGA	< 5 years	Jan/2002-Dec/2003	Netherlands	32-36 wGA	38-41 wGA	HRSV infection	Yes	RSV antigen detection or cell culture
8	Greenberg, 2014	< 2 years	2004-2011	Israel	31-36 wGA	> 36 wGA	HRSV Community-acquired Alveolar Pneumonia	No	Direct immunofluorescence assay
9	Gross, 2017, < 34 wGA	0-14 years	2010-2014	Israel	< 34 wGA	≥ 37 wGA	HRSV infection	Yes	PCR
10	Gross, 2017, 34-36 wGA	0-14 years	2010-2014	Israel	34-36 wGA	≥ 37 wGA	HRSV infection	Yes	PCR
11	Helfrich, 2015, 33–34 wGA	> 90 days	Oct/2005-Apr/2011	USA	33-34 wGA	≥ 37 wGA	HRSV infection	No	International Classification of Diseases code
12	Helfrich, 2015, 35–36 wGA	> 90 days	Oct/2005-Apr/2011	USA	35-36 wGA	≥ 37 wGA	HRSV infection	No	International Classification of Diseases code
13	Horn, 2003, ≤32 wGA	≤ 1 year	Apr/1995-Sep/1996	USA	≤ 32 wGA	≥ 37 wGA	HRSV infection	No	RSV antigen detection and cell culture
14	Horn, 2003, 33–35 wGA	≤ 1 year	Apr/1995-Sep/1996	USA	33-35 wGA	≥ 37 wGA	HRSV infection	No	RSV antigen detection and cell culture
15	Horn, 2003, 36 wGA	≤ 1 year	Apr/1995-Sep/1996	USA	36 wGA	≥ 37 wGA	HRSV infection	No	RSV antigen detection and cell culture
16	Leader, 2003	≤ 1 year	Feb/2000-Apr/2001	USA	33-35 wGA	≥ 37 wGA	HRSV infection	No	Secure patient record
17	Meert, 1990	Unclear/Not reported	1985-1989	USA	≤ 37 wGA	> 37 wGA	HRSV infection	Unclear/Not reported	RSV antigen detection
18	Resch, 2007	Unclear/Not reported	Jan/2001-Jul/2005	Austria	26-36 wGA	≥ 37 wGA	HRSV infection	Yes	ELISA
19	Van De Steen, 2016	≤ 1 year	Oct/2009-Apr/2010 and Oct/2010-Apr/2011	Estonia, Lithuania, Hungary, Slovenia, Croatia, Serbia, Bosnia/Herzegovina, Bulgaria, Czech Republic, Slovakia, Romania, and Ukraine	≤ 36 wGA	≥ 37 wGA	HRSV infection	Yes	Rapid diagnostic test
20	Vo, 2014	≤ 1 year	2010-2012	Canada	Unclear/Not reported	Unclear/Not reported	HRSV infection	Unclear/Not reported	Unclear/Not reported
