# Supplementary material for: Comparison of health care resource utilization among preterm and term infants hospitalized with Human Respiratory Syncytial Virus infections: A systematic review and meta-analysis of retrospective cohort studies
Source: PLoS One. 2020 Feb 21;15(2):e0229357. doi: 10.1371/journal.pone.0229357 (PMC7034889; doi:10.1371/journal.pone.0229357)
Supplement: S6 Table — (PDF) [file pone.0229357.s014.pdf]

1.6. Supplemental Table 6. Individual results of the quality assessment of the included studies using the Newcastle Ottawa scale.

Study ID	Author, year	Selection				Comparability		Outcomes			Study bias
		1) Representativeness of the HRSV hospitalized preterm infants	2) Selection of the HRSV hospitalized term children	3) Ascertainment of gestational age + HRSV infection	4) Demonstration that the use of medical care was not present at the beginning of the study	1) Comparability of preterm and term infants for age at inclusion	2) Comparability of preterm and term infants for a second important factor	1) Assessment of the use of medical care	2) Was follow-up long enough for the use of medical care	3) Complete follow up of all subjects accounted for 80%	
1	Assefa, 2011	0	1	1	1	1	1	1	1	1	Low risk of bias
2	Chi, 2018	1	1	1	1	0	0	1	1	1	Low risk of bias
3	Followell, 2018	0	1	0	1	0	0	1	1	1	High risk of bias
4	Forbes, 2010, ≤32 wGA	1	1	1	1	0	0	1	1	1	Low risk of bias
5	Forbes, 2010, 33–36 wGA	1	1	1	1	0	0	1	1	1	Low risk of bias
6	Gijtenbeek, 2015, < 32 wGA	1	1	1	1	0	1	1	1	1	Low risk of bias
7	Gijtenbeek, 2015, 32-36 wGA	1	1	1	1	0	1	1	1	1	Low risk of bias
8	Greenberg, 2014	1	1	1	1	0	0	1	1	1	Low risk of bias
9	Gross, 2017, < 34 wGA	1	1	1	1	0	0	1	1	1	Low risk of bias
10	Gross, 2017, 34-36 wGA	1	1	1	1	0	0	1	1	1	Low risk of bias
11	Helfrich, 2015, 33–34 wGA	1	1	1	1	1	1	1	1	1	Low risk of bias
12	Helfrich, 2015, 35–36 wGA	1	1	1	1	1	1	1	1	1	Low risk of bias
13	Horn, 2003, ≤32 wGA	1	1	1	1	0	0	1	1	1	Low risk of bias
14	Horn, 2003, 33–35 wGA	1	1	1	1	0	0	1	1	1	Low risk of bias
15	Horn, 2003, 36 wGA	1	1	1	1	0	0	1	1	1	Low risk of bias
16	Leader, 2003	1	1	1	1	0	0	1	1	1	Low risk of bias
17	Meert, 1990	0	1	1	1	0	1	1	1	1	Low risk of bias
18	Resch, 2007	0	1	1	1	0	0	1	1	1	Low risk of bias
19	Van De Steen, 2016	1	1	1	1	0	0	1	1	1	Low risk of bias
20	Vo, 2014	0	1	0	1	0	0	1	1	1	High risk of bias
