# Supplementary material for: Comparison of health care resource utilization among preterm and term infants hospitalized with Human Respiratory Syncytial Virus infections: A systematic review and meta-analysis of retrospective cohort studies
Source: PLoS One. 2020 Feb 21;15(2):e0229357. doi: 10.1371/journal.pone.0229357 (PMC7034889; doi:10.1371/journal.pone.0229357)
Supplement: S7 Table — (PDF) [file pone.0229357.s015.pdf]

1.7. Supplemental table 7. Subgroup analyses of binary outcomes of health care resource utilization among preterm and term infants hospitalized with HRSV infections

Subgroups	RR (95%CI)	95% Prediction interval	N Studies	N preterm infants	N term infants	H¶ (95%CI)	I²§ (95%CI)	P-value heterogeneity	P-value subgroup difference
**Intensive care unit admission**									
**Preterm classification 1**									0.231
Late preterm infants, > 34 wGA	1.5 [0.9 - 2.7]	NA	2	135	852	1.7 [1 - 3.6]	65.8 [0 - 92.2]	0.087	
Preterm, ≤ 34 wGA	2.8 [1.2 - 6.5]	[0 - 71059.3]	3	118	2835	2.9 [1.8 - 4.9]	88.3 [67.7 - 95.8]	< 0.001	
**Preterm classification 2**									0.812
<= 32 wGA	1.6 [1.1 - 2.4]	NA	2	68	2198	1 NA	0 NA	0.342	
> 32 wGA	1.7 [1.3 - 2.3]	[0.9 - 3.5]	5	387	3086	1.4 [1 - 2.3]	47.4 [0 - 80.7]	0.107	
**HRSV Prophylaxis**									0.841
No	2.5 [1.4 - 4.5]	[0.2 - 35]	4	821	8585	3.7 [2.5 - 5.3]	92.5 [84.1 - 96.5]	< 0.001	NA
Yes	2.7 [1.9 - 4]	[0.8 - 9.1]	10	806	4245	2 [1.5 - 2.8]	75.7 [54.9 - 86.9]	< 0.001	
**HRSV detection assay**									0.104
ICD code	2.2 [1.3 - 3.7]	[0.4 - 12]	7	824	5792	3.4 [2.5 - 4.5]	91.2 [84.4 - 95]	< 0.001	
Laboratory detection assays	3.5 [2.9 - 4.2]	[2.3 - 5.3]	4	463	2730	1.5 [1 - 2.6]	57.1 [0 - 85.8]	0.072	
**WHO region**									< 0.001
America	1.8 [1.4 - 2.2]	[1.1 - 2.9]	9	501	5056	1.3 [1 - 2]	43.2 [0 - 73.8]	0.079	
Europe	3.5 [2.9 - 4.2]	[2.6 - 4.7]	5	579	3245	1.3 [1 - 2.2]	43.3 [0 - 79.2]	0.133	
Western Pacific	5.9 [4.6 - 7.5]	NA	1	560	4583	NA	NA	1	
**Emergency department visits**									
**Preterm classification 1**									0.875
Late preterm infants, > 34 wGA	1.1 [0.9 - 1.4]	NA	1	30	215	NA	NA	1	
Preterm, ≤ 34 wGA	1.2 [0.9 - 1.4]	NA	1	28	215	NA	NA	1	
**Preterm classification 2**									0.323
<= 32 wGA	1.2 [0.9 - 1.4]	NA	1	28	215	NA	NA	1	
> 32 wGA	1 [0.8 - 1.3]	NA	2	61	430	1.9 [1 - 3.9]	71.3 [0 - 93.6]	0.062	
**Mechanical ventilation utilization**									

Subgroups	RR (95%CI)	95% Prediction interval	N Studies	N preterm infants	N term infants	H¶ (95%CI)	I2§ (95%CI)	P-value heterogeneity	P-value subgroup difference
**Preterm classification 1**									0.052
Late preterm infants, > 34 wGA	1.1 [0.9 - 1.4]	NA	1	40	102	NA	NA	1	
Preterm, ≤ 34 wGA	0.1 [0 - 1.1]	NA	1	17	7	NA	NA	1	
**Preterm classification 2**									0.052
<= 32 wGA	0.1 [0 - 1.1]	NA	1	17	7	NA	NA	1	
> 32 wGA	1.1 [0.9 - 1.4]	NA	1	40	102	NA	NA	1	
**HRSV Prophylaxis**									0.002
No	15.9 [9.1 - 27.9]	NA	1	560	4583	NA	NA	1	
Yes	1.1 [0.2 - 5.6]	[0 - 1084.1]	4	193	369	2.6 [1.7 - 4.1]	85.3 [63.6 - 94]	< 0.001	
**HRSV detection assay**									< 0.001
ICD code	15.5 [8.9 - 26.9]	NA	2	571	4596	1 NA	0 NA	0.656	
Laboratory detection assays	0.3 [0.1 - 0.8]	NA	2	55	14	1 NA	0 NA	0.414	
**WHO region**									< 0.001
America	5.2 [2.4 - 11.7]	NA	2	138	355	1 NA	0 NA	0.748	
Europe	0.6 [0.3 - 1.4]	[0 - 2478.4]	3	95	116	1.9 [1 - 3.5]	71.7 [4 - 91.6]	0.029	
Western Pacific	15.9 [9.1 - 27.9]	NA	1	560	4583	NA	NA	1	
**Supplemental oxygen utilization**									
**WHO region**									0.459
America	1.4 [0.8 - 2.3]	NA	2	140	396	4.3 [2.5 - 7.6]	94.7 [83.6 - 98.3]	< 0.001	
Europe	1.1 [0.7 - 1.6]	[0 - 118.2]	3	321	1048	3.2 [1.9 - 5.2]	90 [73.2 - 96.2]	< 0.001	
**Case fatality rate**									
**HRSV Prophylaxis**									0.304
No	1.5 [0.1 - 36]	NA	1	248	867	NA	NA	1	
Yes	9.1 [2.4 - 34.8]	NA	1	421	2308	NA	NA	1	

RR: Risk Ratio; N: Number; 95% CI: 95% Confidence Interval; NA: Not Applicable; LOS: Length of stay;

¶H is a measure of the extent of heterogeneity, a value of H =1 indicates homogeneity of effects and a value of H >1indicates a potential heterogeneity of effects.

§: I2 describes the proportion of total variation in study estimates that is due to heterogeneity, a value > 50% indicates presence of heterogeneity
