# Supplementary material for: Comparison of health care resource utilization among preterm and term infants hospitalized with Human Respiratory Syncytial Virus infections: A systematic review and meta-analysis of retrospective cohort studies
Source: PLoS One. 2020 Feb 21;15(2):e0229357. doi: 10.1371/journal.pone.0229357 (PMC7034889; doi:10.1371/journal.pone.0229357)
Supplement: S8 Table — (PDF) [file pone.0229357.s016.pdf]

1.8.Supplemental table 8. Subgroup analyses of continuous outcomes of health care resource utilization among preterm and term infants hospitalized with HRSV infections

Subgroups	SMD (95%CI)	95% Prediction interval	N Studies	N preterm infants	N term infants	H[¶] (95%CI)	I²[§] (95%CI)	P-value heterogeneity	P-value subgroup difference
**Hospitalization LOS**									
**Preterm classification 1**									0,271
Late preterm infants, > 34 wGA	0.4 [0.3 ; 0.5]	NA	2	509	7169	1.0	0.0	0,443	
Preterm, ≤ 34 wGA	0.6 [0.3 ; 0.8]	[-1.5 ; 2.7]	3	85	2205	1.1 [1 - 3.3]	13.2 [0 - 91]	0,316	
**Preterm classification 2**									0,894
<= 32 wGA	0.6 [0.3 ; 0.8]	[-1.5 ; 2.7]	3	85	2205	1.1 [1 - 3.3]	13.2 [0 - 91]	0,316	
> 32 wGA	0.6 [0.4 ; 0.8]	[0 ; 1.2]	6	925	16357	2.1 [1.4 - 3.2]	78.3 [52.3 - 90.2]	0	
**HRSV Prophylaxis**									0,731
No	0.7 [0.4 ; 0.9]	[-0.1 ; 1.4]	6	780	14589	2.2 [1.5 - 3.3]	80.1 [56.9 - 90.8]	0	
Yes	0.5 [0 ; 1.1]	[-6 ; 7.1]	3	321	1048	1.7 [1 - 3.1]	64.3 [0 - 89.8]	0,061	
**HRSV detection assay**									0,371
ICD code	0.5 [0.4 ; 0.7]	[0.1 ; 0.9]	5	904	17910	1.6 [1 - 2.6]	60 [0 - 85]	0,04	
Laboratory detection assays	0.7 [0.4 ; 0.9]	[-0.1 ; 1.4]	7	537	2035	2 [1.4 - 2.9]	74.6 [46 - 88.1]	0,001	
**WHO region**									0,887
America	0.6 [0.5 ; 0.7]	[0.2 ; 1]	9	1120	18897	1.8 [1.3 - 2.5]	68.6 [37 - 84.3]	0,001	
Europe	0.5 [0 ; 1.1]	[-6 ; 7.1]	3	321	1048	1.7 [1 - 3.1]	64.3 [0 - 89.8]	0,061	
**Intensive care unit LOS**									
**Preterm classification 1**									0,559
Late preterm infants, > 34 wGA	0.3 [-0.1 ; 0.7]	NA	2	70	317	1.6 [1 - 3.2]	59 [0 - 90.3]	0,118	
Preterm, ≤ 34 wGA	0.5 [0.2 ; 0.7]	NA	2	68	2198	1.0	0.0	0,971	
**Preterm classification 2**									0,772
<= 32 wGA	0.5 [0.2 ; 0.7]	NA	2	68	2198	1.0	0.0	0,971	
> 32 wGA	0.5 [0.3 ; 0.8]	[-0.6 ; 1.6]	4	274	2515	1.7 [1 - 2.9]	64.5 [0 - 88]	0,038	
**HRSV Prophylaxis**									0,129
No	0.5 [0 ; 1]	[-4.9 ; 5.9]	3	89	645	2.1 [1.1 - 3.7]	76.3 [22.3 - 92.8]	0,015	
Yes	0.9 [0.7 ; 1]	NA	1	266	1034	NA	NA	1	

Subgroups	SMD (95%CI)	95% Prediction interval	N Studies	N preterm infants	N term infants	H¶ (95%CI)	I²§ (95%CI)	P-value heterogeneity	P-value subgroup difference
**HRSV detection assay**									0.621
ICD code	0.5 [0.4 ; 0.6]	NA	2	213	3966	1 NA	0 NA	0,728	
Laboratory detection assays	0.6 [0.2 ; 1]	[-1 ; 2.2]	4	355	1679	2.3 [1.4 - 3.7]	81.4 [51.7 - 92.9]	0,001	
**WHO region**									0.197
America	0.5 [0.3 ; 0.7]	[-0.1 ; 1.1]	5	302	4611	1.5 [1 - 2.4]	53.4 [0 - 82.8]	0,072	
Europe	0.7 [0.4 ; 1.1]	NA	2	306	1136	1.7 [1 - 3.5]	64 [0 - 91.8]	0,096	
**Age at time of index HRSV infection**									
**Preterm classification 1**									0,063
Late preterm infants, > 34 wGA	0.0 [-0.0 ; 0.1]	NA	1	479	6954	NA	NA	1	
Preterm, ≤ 34 wGA	0.4 [0.0 ; 0.7]	NA	1	40	1983	NA	NA	1	
**Preterm classification 2**									0,047
<= 32 wGA	0.4 [0.0 ; 0.7]	NA	1	40	1983	NA	NA	1	
> 32 wGA	0.0 [-0.0 ; 0.1]	[-0.1 ; 0.2]	4	864	15927	1 [1 - 1.4]	0 [0 - 45.9]	0,838	

SMD: Standardised Mean Difference; N: Number; 95% CI: 95% Confidence Interval; NA: Not Applicable; LOS: Length of stay;

¶H is a measure of the extent of heterogeneity, a value of H =1 indicates homogeneity of effects and a value of H >1indicates a potential heterogeneity of effects.

§: I2 describes the proportion of total variation in study estimates that is due to heterogeneity, a value > 50% indicates presence of heterogeneity
