# Supplementary material for: Comparison of health care resource utilization among preterm and term infants hospitalized with Human Respiratory Syncytial Virus infections: A systematic review and meta-analysis of retrospective cohort studies
Source: PLoS One. 2020 Feb 21;15(2):e0229357. doi: 10.1371/journal.pone.0229357 (PMC7034889; doi:10.1371/journal.pone.0229357)
Supplement: S9 Table — (PDF) [file pone.0229357.s017.pdf]

1.9. Supplemental table 9. P-value of Khi-2 and Fisher exact tests for qualitative confounding factors

			Data extracted from included studies				Results from this study		
**Author, year**	**Author of included study definitions**	**This study definitions**	**Total preterm**	**Number in preterm**	**Total term**	**Number in term**	**P-value Khi-2 test**	**P-value Fisher exact test**	**Status**
Assefa, 2011	Gender (male)	Gender (male)	11	6	13	7	1.000	1.000	Symmetric
Forbes, 2010, ≤32 wGA	Respiratory comorbid conditions	Respiratory comorbid conditions	40	17	1983	4	0.198	0.194	Symmetric
Forbes, 2010, ≤32 wGA	Male infants	Gender (male)	40	28	1983	1162	< 0.001	< 0.001	Asymmetric
Forbes, 2010, ≤32 wGA	CHD or other cardiovascular comorbid conditions	**Heart disease**	40	19	1983	67	< 0.001	< 0.001	Asymmetric

Author, year	Author of included study definitions	This study definitions	Data extracted from included studies				Results from this study		
			Total preterm	Number in preterm	Total term	Number in term	P-value Khi-2 test	P-value Fisher exact test	Status
Forbes, 2010, ≤32 wGA	All other comrbid conditions	Underlying disease	40	10	1983	20	< 0.001	< 0.001	Asymmetric
Forbes, 2010, 33–36 wGA	Respiratory comorbid conditions	Respiratory comorbid conditions	173	5	1983	4	0.003	0.002	Asymmetric
Forbes, 2010, 33–36 wGA	Male infants	Gender (male)	173	122	1983	1162	< 0.001	< 0.001	Asymmetric
Forbes, 2010, 33–36 wGA	CHD or other cardiovascular comorbid conditions	Heart disease	173	30	1983	67	< 0.001	< 0.001	Asymmetric
Forbes, 2010, 33–36 wGA	All other comrbid conditions	Underlying disease	173	16	1983	20	< 0.001	< 0.001	Asymmetric
Gijtenbeek, 2015, GA 32–36 wGA	Day-care attendance 1st year	Day-care attendance 1st year	38	9	926	249	0.802	0.852	Symmetric
Gijtenbeek, 2015, GA 32–36 wGA	Male gender	Gender (male)	38	25	926	526	0.352	0.317	Symmetric
Gijtenbeek, 2015, GA 32–36 wGA	Passive smoking 1st year of life	Passive smoking 1st year of life	38	5	926	46	0.066	0.045	Asymmetric
Gijtenbeek, 2015, GA 32–36 wGA	School age siblings >= 1	School age siblings >= 1	38	5	926	135	0.993	1.000	Symmetric
Gijtenbeek, 2015, GA 32–36 wGA	Smoking while pregnant	Smoking while pregnant	38	8	926	180	0.970	0.834	Symmetric
Gijtenbeek, 2015, GA 32–36 wGA	Twins	Twins	38	14	926	260	0.322	0.271	Symmetric
Gijtenbeek, 2015, GA 32–36 wGA	Age <= 3 months before/during RSV season	Age <= 3 months before/during RSV season	38	25	926	636	0.843	0.723	Symmetric
Gijtenbeek, 2015, GA 32–36 wGA	Asthma in family history	Asthma in family history	38	17	926	318	0.252	0.223	Symmetric
Gijtenbeek, 2015, GA 32–36 wGA	Breastfeeding <2 months	Breastfeeding <2 months	38	20	926	399	0.319	0.248	Symmetric
Greenberg, 2014	Males	Gender (male)	116	66	515	265	0.339	0.305	Symmetric
Greenberg, 2014	Underlying disease	Underlying disease	116	25	515	70	0.043	0.043	Asymmetric
Gross, 2017, < 34	Coinfection with other viruses	Coinfection with other	50	7	637	69	0.650	0.482	Symmetric

Author, year	Author of included study definitions	This study definitions	Data extracted from included studies				Results from this study		
			Total preterm	Number in preterm	Total term	Number in term	P-value Khi-2 test	P-value Fisher exact test	Status
wGA		viruses							
Gross, 2017, < 34 wGA	Gender, Male	Gender (male)	50	33	637	364	0.284	0.238	Symmetric
Gross, 2017, 34-36 wGA	Coinfection with other viruses	Coinfection with other viruses	105	14	637	69	0.558	0.503	Symmetric
Gross, 2017, 34-36 wGA	Gender, Male	Gender (male)	105	58	637	364	0.796	0.750	Symmetric
Helfrich, 2015, 33–34 wGA	Males with RSV Hospitalization	Gender (male)	5938	84	573645	3959	< 0.001	< 0.001	Asymmetric
Helfrich, 2015, 35–36 wGA	Males with RSV Hospitalization	Gender (male)	19952	278	573645	3959	< 0.001	< 0.001	Asymmetric
Horn, 2003, ≤32 wGA	Bronchopulmonary dysplasia	Bronchopulmonary dysplasia	28	1	215	5	1.000	0.524	Symmetric
Horn, 2003, ≤32 wGA	Heart disease	Heart disease	28	1	215	17	0.660	0.703	Symmetric
Horn, 2003, 33–35 wGA	Bronchopulmonary dysplasia	Bronchopulmonary dysplasia	31	2	215	5	0.475	0.216	Symmetric
Horn, 2003, 33–35 wGA	Heart disease	Heart disease	31	4	215	17	0.557	0.315	Symmetric
Horn, 2003, 36 wGA	Bronchopulmonary dysplasia	Bronchopulmonary dysplasia	30	2	215	5	0.452	0.206	Symmetric
Horn, 2003, 36 wGA	Heart disease	Heart disease	30	3	215	17	0.971	0.720	Symmetric
Leader, 2003	Sex, Male	Gender (male)	48	28	36	16	0.298	0.271	Symmetric
Resch, 2007	Bacterial co-infection	Bacterial co-infection	42	4	422	13	0.091	0.058	Symmetric
Van De Steen, 2016	Bronchopulmonary dysplasia	Bronchopulmonary dysplasia	266	41	1034	0	< 0.001	< 0.001	Asymmetric
Van De Steen, 2016	Male	Gender (male)	266	147	1034	591	0.627	0.580	Symmetric
Van De Steen, 2016	Congenital heart disease	Heart disease	266	27	1034	0	< 0.001	< 0.001	Asymmetric
Van De Steen, 2016	Any chronic disease	Underlying disease	266	91	1034	0	< 0.001	< 0.001	Asymmetric
