# Supplementary material for: Comparison of health care resource utilization among preterm and term infants hospitalized with Human Respiratory Syncytial Virus infections: A systematic review and meta-analysis of retrospective cohort studies
Source: PLoS One. 2020 Feb 21;15(2):e0229357. doi: 10.1371/journal.pone.0229357 (PMC7034889; doi:10.1371/journal.pone.0229357)
Supplement: S10 Table — (PDF) [file pone.0229357.s018.pdf]

1.10. Supplemental table 10. Sensitivity analyses of binary outcomes of the symmetrically distributed confounding factors

Subgroups	RR (95%CI)	95% Prediction interval	N Studies	N preterm infants	N term infants	H (95%CI)	I² (95%CI)	P heterogeneity
**Intensive care unit admission**								
**Gender (male)**								
Symmetric	3.1 [2.0 - 4.8]	[0.8 - 11.7]	7	636	4855	1.9 [1.3 - 2.8]	71.9 [39 - 87]	0,002
**Heart disease**								
Symmetric	1.5 [1.1 - 1.9]	[0.2 - 9.2]	3	89	645	1 [1 - 2.9]	0 [0 - 87.8]	0,428
**School age siblings > 1**								
Symmetric	15.3 [1 - 242.8]	NA	1	11	13	NA	NA	1
**Smoking while pregnant**								
Symmetric	15.3 [1 - 242.8]	NA	1	11	13	NA	NA	1
**Twins**								
Symmetric	15.3 [1 - 242.8]	NA	1	11	13	NA	NA	1
**Age < 3 months before_during RSV season**								
Symmetric	15.3 [1 - 242.8]	NA	1	11	13	NA	NA	1
**Asthma in family history**								
Symmetric	15.3 [1 - 242.8]	NA	1	11	13	NA	NA	1
**Breastfeeding < 2 months**								
Symmetric	15.3 [1 - 242.8]	NA	1	11	13	NA	NA	1
**Coinfection with other viruses**								
Symmetric	1.6 [1.2 - 2.2]	NA	2	59	430	1 NA	0 NA	0,536
**Bronchopulmonary dysplasia**								
Symmetric	1.2 [0.3 - 5.1]	NA	1	13	54	NA	NA	1
**Bacterial co-infection**								
Symmetric	8 [4.1 - 15.6]	NA	1	50	637	NA	NA	1
**Emergency department visits**								
**Heart disease**								
Symmetric	1.1 [0.9 - 1.2]	[0.4 - 2.7]	3	89	645	1.5 [1 - 2.8]	54 [0 - 86.8]	0,114
**Coinfection with other viruses**								
Symmetric	1 [0.8 - 1.3]	NA	2	59	430	2 [1 - 4.2]	74.8 [0 - 94.3]	0,046

Subgroups	RR (95%CI)	95% Prediction interval	N Studies	N preterm infants	N term infants	H (95%CI)	I² (95%CI)	P heterogeneity
**Mechanical ventilation utilization**								
**Gender (male)**								
Symmetric	0.8 [0.2 - 4.2]	NA	2	49	20	2 [1 - 4.2]	74.5 [0 - 94.2]	0,048
**Day-care attendance 1st year**								
Symmetric	0.4 [0.1 - 1.1]	NA	1	38	7	NA	NA	1
**School age siblings > 1**								
Symmetric	8.2 [0.5 - 143]	NA	1	11	13	NA	NA	1
**Smoking while pregnant**								
Symmetric	8.2 [0.5 - 143]	NA	1	11	13	NA	NA	1
**Twins**								
Symmetric	8.2 [0.5 - 143]	NA	1	11	13	NA	NA	1
**Age < 3 months before_during RSV season**								
Symmetric	8.2 [0.5 - 143]	NA	1	11	13	NA	NA	1
**Asthma in family history**								
Symmetric	8.2 [0.5 - 143]	NA	1	11	13	NA	NA	1
**Breastfeeding < 2 months**								
Symmetric	8.2 [0.5 - 143]	NA	1	11	13	NA	NA	1
**Bronchopulmonary dysplasia**								
Symmetric	1.1 [0.9 - 1.3]	NA	2	57	109	1.9 [1 - 4.1]	73.4 [0 - 94]	0,052
**Supplemental oxygen utilization**								
**Gender (male)**								
Symmetric	1.1 [0.6 - 1.9]	NA	2	304	1041	3.8 [2.1 - 7]	93.2 [77.8 - 97.9]	0
**Day-care attendance 1st year**								
Symmetric	0.7 [0.5 - 1]	NA	1	38	7	NA	NA	1
**Bronchopulmonary dysplasia**								
Symmetric	1 [0.8 - 1.1]	NA	2	30	61	1 NA	0 NA	0,991
**Case fatality rate**								
**Gender (male)**								

Subgroups	RR (95%CI)	95% Prediction interval	N Studies	N preterm infants	N term infants	H (95%CI)	I² (95%CI)	P heterogeneity
Symmetric	6.9 [2 - 23.8]	NA	2	669	3175	1 NA	5.2 NA	0,304

RR: Risk Ratio; N: Number; 95% CI: 95% Confidence Interval; NA: Not Applicable; LOS: Length of stay;

¶H is a measure of the extent of heterogeneity, a value of H =1 indicates homogeneity of effects and a value of H >1indicates a potential heterogeneity of effects.

§: I2 describes the proportion of total variation in study estimates that is due to heterogeneity, a value > 50% indicates presence of heterogeneity
