# Supplementary material for: Comparison of health care resource utilization among preterm and term infants hospitalized with Human Respiratory Syncytial Virus infections: A systematic review and meta-analysis of retrospective cohort studies
Source: PLoS One. 2020 Feb 21;15(2):e0229357. doi: 10.1371/journal.pone.0229357 (PMC7034889; doi:10.1371/journal.pone.0229357)
Supplement: S11 Table — (PDF) [file pone.0229357.s019.pdf]

1.11. Supplemental table 11. Sensitivity analyses of continuous outcomes of the symmetrically distributed confounding factors

Subgroups	SMD (95%CI)	95% Prediction interval	N Studies	N preterm infants	N term infants	H (95%CI)	I² (95%CI)	P heterogeneity
**Hospitalization LOS**								
**Gender (male)**								
Symmetric	0.6 [0.2 ; 0.9]	[-0.8 ; 2]	4	392	3060	1.9 [1.2 - 3.2]	73.3 [24.9 - 90.5]	0,011
**Heart disease**								
Symmetric	0.8 [0.2 ; 1.3]	[-5.7 ; 7.2]	3	89	645	2.4 [1.3 - 4.1]	82.1 [44.7 - 94.2]	0,004
**Day-care attendance 1st year**								
Symmetric	0.0 [-0.8 ; 0.8]	NA	1	38	7	NA	NA	1
**Coinfection with other viruses**								
Symmetric	1.0 [0.6 ; 1.4]	NA	2	59	430	1.3	43.4	0,184
**Bronchopulmonary dysplasia**								
Symmetric	0.4 [-0.5 ; 1.3]	NA	1	17	7	NA	NA	1
**Intensive care unit LOS**								
**Gender (male)**								
Symmetric	0.7 [0.3 ; 1.1]	NA	2	306	3017	2.3 [1.1 - 4.8]	81.4 [21.2 - 95.6]	0,02
**Heart disease**								
Symmetric	0.5 [0.0 ; 1.0]	[-4.9 ; 5.9]	3	89	645	2.1 [1.1 - 3.7]	76.3 [22.3 - 92.8]	0,015
**Coinfection with other viruses**								
Symmetric	0.7 [0.3 ; 1.1]	NA	2	59	430	1.6 [1 - 3.2]	58.8 [0 - 90.3]	0,119
**Bronchopulmonary dysplasia**								
Symmetric	0.5 [0.0 ; 1.0]	[-4.9 ; 5.9]	3	89	645	2.1 [1.1 - 3.7]	76.3 [22.3 - 92.8]	0,015

Subgroups	SMD (95%CI)	95% Prediction interval	N Studies	N preterm infants	N term infants	H (95%CI)	I² (95%CI)	P heterogeneity
**Age at time of index HRSV infection**								
**Gender (male)**								
Symmetric	0.2 [-0.2 ; 0.6]	NA	2	88	2019	1.5 [1 - 3.1]	56.8 [0 - 89.6]	0,128

SMD: Standardised Mean Difference; N: Number; 95% CI: 95% Confidence Interval; NA: Not Applicable; LOS: Length of stay;

¶H is a measure of the extent of heterogeneity, a value of H =1 indicates homogeneity of effects and a value of H >1indicates a potential heterogeneity of effects.

§: I2 describes the proportion of total variation in study estimates that is due to heterogeneity, a value > 50% indicates presence of heterogeneity
