# Supplementary material for: Comparison of health care resource utilization among preterm and term infants hospitalized with Human Respiratory Syncytial Virus infections: A systematic review and meta-analysis of retrospective cohort studies
Source: PLoS One. 2020 Feb 21;15(2):e0229357. doi: 10.1371/journal.pone.0229357 (PMC7034889; doi:10.1371/journal.pone.0229357)
Supplement: Attachment — Submitted filename: PONE-D-19-26852_reviewer.pdf [file pone.0229357.s020.pdf]

PLOS ONE

Comparison of health care resource utilization among preterm and term infants hospitalized with HRSV infections: the HARIPI systematic review and meta-analysis of retrospective cohort studies.

--Manuscript Draft--

**Manuscript Number:**	PONE-D-19-26852
**Article Type:**	Research Article
**Full Title:**	Comparison of health care resource utilization among preterm and term infants hospitalized with HRSV infections: the HARIPI systematic review and meta-analysis of retrospective cohort studies.
**Short Title:**	Preterm birth and health care resource utilization for HRSV infection.
**Corresponding Author:**	Richard Njouom, PhD<br>1Centre Pasteur du Cameroun, Yaounde, Cameroon<br>Yaounde, Centre CAMEROON
**Keywords:**	HRSV infection; health care resource utilization; Preterm birth
**Abstract:**	Introduction Data on the variation in the medical resource utilization rate of HRSV-infected children by gestational age have recently been made available. This review aimed to determine whether prematurity is independently associated with the use of medical resources in hospitalized children for HRSV infections.<br>Methods We conducted this systematic review on cohort studies published on the medical resources use in preterm and full-term patients hospitalized for confirmed HRSV infections. We searched PubMed, Embase, and Global Index medicus for eligible studies. The standardized mean difference (SMD) and Risk Ratio (RR) with their 95% confidence intervals (95% CI) were estimated as summary statistics with random effects meta-analysis. The overall results were adjusted to the common confounders by stratified analyses.<br>Results A total of 14 articles (20 studies) were included. Compared to full-term, preterm hospitalized with HRSV infections had more frequent intensive care unit admission (RR = 2.6, 95% CI = 1.9-3.5), increased length of stay in hospital (SMD = 0.6, 95% CI = 0.5-0.8) and ICU (SMD = 0.6, 95% CI = 0.4-0.8) and increased case fatality rate (RR = 6.9, 95% CI = 2.0-23.8). Mechanical ventilation utilization were more frequent in preterm children ≤ 2 years (RR = 15.5, 95% CI = 8.9-26.4) and those who did not receive prophylaxis against HRSV (RR = 15.9, 95% CI = 9.1-27.9)] than in full-term children. No differences were identified in the frequency of emergency department visits, oxygen utilization, and the age at the first HRSV episode between preterm and full-term infants.<br>Conclusions Regardless of gestational age, preterm infants hospitalized for HRSV infections have an increased frequency of use of health resources and poor outcomes compared to full-term infants. HRSV vaccine development programs for pregnant women should be accelerated.<br><br>Review registration PROSPERO, CRD42019124375.
**Order of Authors:**	Sebastien Kenmoe
	Cyprien Kengne-Nde
	Abdou Fatawou Modiyinji
	Giuseppina La Rosa
	Richard Njouom, PhD
**Additional Information:**	
**Question**	**Response**
**Financial Disclosure**<br><br>Enter a financial disclosure statement that	The author(s) received no specific funding for this work.

Powered by Editorial Manager® and ProduXion Manager® from Aries Systems Corporation

describes the sources of funding for the work included in this submission. Review the submission guidelines for detailed requirements. View published research articles from *PLOS ONE* for specific examples.<br><br>This statement is required for submission and **will appear in the published article** if the submission is accepted. Please make sure it is accurate.<br><br>**Unfunded studies**<br>Enter: *The author(s) received no specific funding for this work.*<br><br>**Funded studies**<br>Enter a statement with the following details:<br>• Initials of the authors who received each award<br>• Grant numbers awarded to each author<br>• The full name of each funder<br>• URL of each funder website<br>• Did the sponsors or funders play any role in the study design, data collection and analysis, decision to publish, or preparation of the manuscript?<br>• **NO** - Include this sentence at the end of your statement: *The funders had no role in study design, data collection and analysis, decision to publish, or preparation of the manuscript.*<br>• **YES** - Specify the role(s) played.<br><br>* typeset	
**Competing Interests**<br><br>Use the instructions below to enter a competing interest statement for this submission. On behalf of all authors, disclose any competing interests that could be perceived to bias this work—acknowledging all financial support and any other relevant financial or non-financial competing interests.<br><br>This statement **will appear in the published article** if the submission is accepted. Please make sure it is accurate. View published research articles from *PLOS ONE* for specific examples.	The authors have declared that no competing interests exist.

Powered by Editorial Manager® and ProduXion Manager® from Aries Systems Corporation

**NO authors have competing interests**<br><br>Enter: *The authors have declared that no competing interests exist.*<br><br>**Authors with competing interests**<br><br>Enter competing interest details beginning with this statement:<br><br>*I have read the journal's policy and the authors of this manuscript have the following competing interests: [insert competing interests here]*<br><br>* typeset	
**Ethics Statement**<br><br>Enter an ethics statement for this submission. This statement is required if the study involved:<br><br>• Human participants<br>• Human specimens or tissue<br>• Vertebrate animals or cephalopods<br>• Vertebrate embryos or tissues<br>• Field research<br><br>Write "N/A" if the submission does not require an ethics statement.<br><br>General guidance is provided below. Consult the submission guidelines for detailed instructions. **Make sure that all information entered here is included in the Methods section of the manuscript.**	This review reports previously published data and ethical clearance was not required. The protocol for this review has been registered in the PROSPERO database under number CRD42018116067.

Powered by Editorial Manager® and ProduXion Manager® from Aries Systems Corporation

**Format for specific study types**<br><br>**Human Subject Research (involving human participants and/or tissue)**<br>• Give the name of the institutional review board or ethics committee that approved the study<br>• Include the approval number and/or a statement indicating approval of this research<br>• Indicate the form of consent obtained (written/oral) or the reason that consent was not obtained (e.g. the data were analyzed anonymously)<br><br>**Animal Research (involving vertebrate animals, embryos or tissues)**<br>• Provide the name of the Institutional Animal Care and Use Committee (IACUC) or other relevant ethics board that reviewed the study protocol, and indicate whether they approved this research or granted a formal waiver of ethical approval<br>• Include an approval number if one was obtained<br>• If the study involved *non-human primates*, add *additional details* about animal welfare and steps taken to ameliorate suffering<br>• If anesthesia, euthanasia, or any kind of animal sacrifice is part of the study, include briefly which substances and/or methods were applied<br><br>**Field Research**<br><br>Include the following details if this study involves the collection of plant, animal, or other materials from a natural setting:<br>• Field permit number<br>• Name of the institution or relevant body that granted permission	
**Data Availability**<br><br>Authors are required to make all data underlying the findings described fully available, without restriction, and from the time of publication. PLOS allows rare exceptions to address legal and ethical concerns. See the PLOS Data Policy and FAQ for detailed information.	Yes - all data are fully available without restriction

Powered by Editorial Manager® and ProduXion Manager® from Aries Systems Corporation

A Data Availability Statement describing where the data can be found is required at submission. Your answers to this question constitute the Data Availability Statement and **will be published in the article**, if accepted.<br><br>**Important:** Stating 'data available on request from the author' is not sufficient. If your data are only available upon request, select 'No' for the first question and explain your exceptional situation in the text box.<br><br>Do the authors confirm that all data underlying the findings described in their manuscript are fully available without restriction?	
**Describe where the data may be found in full sentences. If you are copying our sample text, replace any instances of XXX with the appropriate details.**<br><br>• If the data are **held or will be held in a public repository**, include URLs, accession numbers or DOIs. If this information will only be available after acceptance, indicate this by ticking the box below. For example: *All XXX files are available from the XXX database (accession number(s) XXX, XXX.*).<br>• If the data are all contained **within the manuscript and/or Supporting Information files**, enter the following: *All relevant data are within the manuscript and its Supporting Information files.*<br>• If neither of these applies but you are able to provide **details of access elsewhere**, with or without limitations, please do so. For example:<br><br>*Data cannot be shared publicly because of [XXX]. Data are available from the XXX Institutional Data Access / Ethics Committee (contact via XXX) for researchers who meet the criteria for access to confidential data.*<br><br>*The data underlying the results presented in the study are available from (include the name of the third party*	All relevant data are within the manuscript and its Supporting Information files.

Powered by Editorial Manager® and ProduXion Manager® from Aries Systems Corporation

and contact information or URL).<br>• This text is appropriate if the data are owned by a third party and authors do not have permission to share the data.<br><br>* typeset	
Additional data availability information:	Tick here if your circumstances are not covered by the questions above and you need the journal’s help to make your data available.

Manuscript
Click here to download Manuscript Manuscript_Preterm vs Term_PO.docx

Comparison of health care resource utilization among preterm and term infants hospitalized with HRSV infections: the HARIPI systematic review and meta-analysis of retrospective cohort studies

Sebastien Kenmoe[1], PhD, Cyprien Kengne-Nde[2], MSc, Abdou Fatawou Modiyinji[1,3], MSc, Giuseppina La Rosa[4], MSc, Richard Njouom[1*], PhD

1: Department of Virology, Centre Pasteur of Cameroon, 451 Rue 2005, P.O. Box 1274 Yaoundé, Cameroon

2: National AIDS Control Committee, Epidemiological Surveillance, Evaluation and Research Unit, P.O. Box 1459Yaounde, Cameroon

3: Department of Animals Biology and Physiology, Faculty of Sciences, University of Yaoundé I, P.O. Box 337, Yaoundé, Cameroon

4: Italian National Institute of Health, Viale Regina Elena, 299-00161 Rome, Italy

* Correspondence to: Richard Njouom, PhD/HDR

Centre Pasteur Cameroon, P.O Box: 1274 Yaoundé

Email: njouom@pasteur-yaounde.org / njouom@yahoo.com

Telephone: Office: (00237) 222 23 10 15 and mobile: (00237) 699 65 47 67

Abstract

Introduction Data on the variation in the medical resource utilization rate of HRSV-infected children by gestational age have recently been made available. This review aimed to determine whether prematurity is independently associated with the use of medical resources in hospitalized children for HRSV infections.

Methods We conducted this systematic review on cohort studies published on the medical resources use in preterm and full-term patients hospitalized for confirmed HRSV infections. We searched PubMed, Embase, and Global Index medicus for eligible studies. The standardized mean difference (SMD) and Risk Ratio (RR) with their 95% confidence intervals (95% CI) were estimated as summary statistics with random effects meta-analysis. The overall results were adjusted to the common confounders by stratified analyses.

Results A total of 14 articles (20 studies) were included. Compared to full-term, preterm hospitalized with HRSV infections had more frequent intensive care unit admission (RR = 2.6, 95% CI = 1.9-3.5), increased length of stay in hospital (SMD = 0.6, 95% CI = 0.5-0.8) and ICU (SMD = 0.6, 95% CI = 0.4-0.8) and increased case fatality rate (RR = 6.9, 95% CI = 2.0-23.8). Mechanical ventilation utilization were more frequent in preterm children ≤ 2 years (RR = 15.5, 95% CI = 8.9-26.4) and those who did not receive prophylaxis against HRSV (RR = 15.9, 95% CI = 9.1-27.9)] than in full-term children. No differences were identified in the frequency of emergency department visits, oxygen utilization, and the age at the first HRSV episode between preterm and full-term infants.

Conclusions Regardless of gestational age, preterm infants hospitalized for HRSV infections have an increased frequency of use of health resources and poor outcomes compared to full-term infants. HRSV vaccine development programs for pregnant women should be accelerated.

Review registration PROSPERO, CRD42019124375.

Keywords: HRSV infection, health care resource utilization, prematurity.

Introduction

Human Respiratory syncytial virus (HRSV) is one of the leading causes of acute lower respiratory infection in children worldwide [1–3]. Almost all children are exposed at least once to HRSV at 2 years old, with infection peak occurring between 2-6 months of life [4]. HRSV does not induce long lasting immunity and is therefore a common cause of reinfection [4]. HRSV severe infections are responsible for hospitalization, Intensive Care Unit (ICU) admission, Emergency Department (ED) visits, and the use of Mechanical Ventilation (MV) and/or oxygen supplementation registered mostly in children less than 1 year of age [5–9]. In addition to this burden the HRSV is also responsible for a high Case Fatality Rate (CFR) in resource-constrained countries [2]. HRSV infections are also responsible for a substantial economic burden on society in terms of the costs of palivizumab immunoprophylaxis, hospitalization costs, and productivity and time lost [10–12]. Several risk factors for infection, severity and mortality of HRSV infections in infants have been identified [13,14]. The most important included low birth weight, young age before or during the HRSV season, immunodeficiency, presence of other conditions (chronic lung diseases, bronchopneumonia disease...), multiple birth, history of atopy, tobacco exposure, male gender, breastfeeding within 2 months or less, overcrowding with school-age siblings or daycare attendance and prematurely infants [14]. Studies have shown increased risk of hospitalization in preterm (PT) compared to full-term (FT) infants with this hospitalization rate inversely correlated with decreased gestational age [15]. There is no antiviral or effective vaccine against HRSV and the management is mainly based on symptom and prevention of complications [16–19]. Palivizumab is a humanized monoclonal antibody used for preventative or prophylactic action against HRSV for the reduction of rates and duration of hospitalization related to severe HRSV infections in high-risk children [children with bronchopneumonia disease and/or children born less than 32 weeks of gestational age (wGA)]

[20–22]. However, prophylaxis with palivizumab is recommended only for vulnerable children because of its high cost. The cost-effectiveness analysis for palivizumab allows regular review of palivizumab administration guidelines [23]. The 2014 guidelines issued restrictions on certain categories of infants (infants 29-35 wGA without comorbidities). Controversial data has been reported according to increased morbidity following 2014 guidelines restrictions in the administration of palivizumab [24–27]. Studies also reported an increased risk of hospitalization with HRSV in late PT (34 to <37 wGA) who however are not eligible to receive palivizumab prophylaxis [28]. To date, the synthesis of published data comparing the morbidity of HRSV infections in PT (early and late PT) and FT infants remains to be done. These summary data from the comparison of the consequences of premature birth on HRSV infections are needed to further inform the public health stakeholders about the administration guidelines of palivizumab. The objectives of this review were to compare the use of health care resources related to HRSV infections in PT and FT patients.

Methods

Study design

This study was part of the HARIPI (HRSV Acute Respiratory Infections in Preterm Infants) systematic review and meta-analysis that aimed to describe the burden of HRSV in PT infants. The PRISMA guidelines were followed for the writing of this review (Supplementary table 1) [29]. The protocol of this systematic summary was published in the PROSPERO database under registration number CRD42019124375. This systematic review is based on published studies with participants who have been disidentified and comply with research ethics standards and therefore the approval of an ethics committee was not necessary.

Inclusion criteria

Studies reporting cohorts of patients of all ages hospitalized with confirmed HRSV infections were included. We adopted the classification mode of PT infants proposed by Engle et al. (PT, ≤ 34 wGA and late PT,> 34 wGA) and a second classification defined in this review (≤ 32 wGA vs > 32 wGA) [30]. This second gestational age classification mode considered in this work was consistent with the 32 wGA threshold considered for eligibility to Palivizumab administration in children <1 year of age born between 29 and 32 wGA with other chronic pulmonary comorbidities recommended by the American Academy of Pediatrics in 2014 [22]. The diagnosis of HRSV infection was considered for viral laboratory detection conducted during the study or data recorded in databases or patient files. A second group of term patients (≥37 WGA) hospitalized with HRSV infections were the comparator group. Studies enrolling only patients with comorbidity (congenital malformations, low birth weight or immunocompromised) were excluded to reduce potential confounding effects. The frequency of health resources use was compared between the 2 groups (PT vs FT infants) by examining variables such as admission to ICU, ED visits. Prospective and retrospective cohort studies were included without linguistic, temporal or geographic restrictions.

Search strategy

The articles published until July 22, 2019 were consulted by an electronic search in the Pubmed, Embase, and Global Index medicus databases. The research equation focused on premature birth, HRSV and health care utilization (Supplementary table 2). Additional articles were obtained by manual search in the references list of relevant review and included articles.

Study selection

We used the EndNote Reference Manager software to remove duplicates. Titles and article abstracts were independently analyzed and selected by two authors (SK and AFM). The disagreements were resolved by discussion between the authors and a third referee (RN).

Data extraction

We (SK and AFM) extracted article related data such as: first author, year of publication, study design, recruitment period, timing of data collection, and country. We collected participant related data such as: age at inclusion, gestational age, used of prophylaxis against HRSV, indicators of hospitalization severity such as length of stay (LOS), admission to the ICU and/or use of MV, ED visits, and HRSV detection assay used. The data collected for the use of health care resources was the total of PT and the total of FT and the number presenting the outcome for each group. The data collected for the confounding factors were the total numbers of PT and FT infant and the numbers presenting the confounding factor in each group.

Risk of bias assessment

The included studies were independently evaluated by two authors (SK and AFM) at the methodological level using the Newcastle Ottawa Scale for non-randomised comparative studies [31] (Supplementary table 3). Newcastle Ottawa for cohort studies is a 13-star scale that evaluates the selection of participants (6 stars), the comparability of groups (2 stars) and the outcomes (5 stars).

Statistical analysis

We used meta-analyses for binary data (metabin) and continuous data (metacont) in the R software version 3.5.1 [32,33]. For a minimum of three studies, these functions allow to estimate all the parameters that are important for the meta-analyses (effect, heterogeneity and publication bias). This is the reason why we considered for our review, only the outcomes with at least three studies. Data from included studies (numbers, means, and standard deviation) were used to evaluate risk ratio (RR), standardised mean difference (SMD) with their 95% CIs using a random effect model [34]. We chose RR rather than OR because this parameter is indicated in measuring effect for prospective studies with binary outcomes [35]. We used the SMD rather than the mean difference because this parameter is more generalizable [36]. Means and standard deviations were estimated for studies in which they were not provided using the method described by Wan et al [37]. Differences between groups were considered statistically significant for values of $p < 0.05$. Heterogeneity across included studies was assessed using the I^2 values from the Q test statistic for which a value > 50% was considered to imply heterogeneity. Subgroup analyses including gestational age, use of HRSV prophylaxis, HRSV detection assays (ICD code vs laboratory detection assay), and WHO region were done to investigate the possible sources of heterogeneity. We used the symmetry of funnel plots and did the Egger test to assess the presence of publication and selective reporting bias [38]. A p-value < 0.1 was considered indicative of statistically significant publication bias. The robustness of the results was estimated through sensitivity analyses including only studies with a low risk of bias and patients ≤ 2 years. We evaluated the influence of confounding factors on overall findings by stratified analyses, including bronchopulmonary dysplasia, HRSV co-detection with other viruses or bacteria, presence of other underlying medical conditions, breastfeeding, crowding, smoke exposure, age before/during HRSV season, male gender, multiple gestation, and asthma in family

history. For each potential confounding factor, we used the total numbers of PT and FT infants and the number with the confounding factor in the PT and FT group from the included studies to recalculate the p-value using the Khi-2 and Fisher exact tests. For Fisher exact or Khi-2 p-value <0.05, we considered the confounding factor to have an asymmetric distribution between PT and FT infant and symmetric if else. To appreciate the effect when the confounding factor is controlled, we conducted a sensitivity analysis with included studies with a symmetric distribution of cofounders between PT and FT infants.

Results

Study selection and characteristics

Our search strategy found 3361 articles through database searching and 7 through other sources and we removed 829 duplicates (Figure 1). We consulted the titles and abstracts of 2519 articles and eliminated 2444 irrelevant according to inclusion criteria of the study. We consulted the full text of 75 articles and eliminated 64 for multiple reasons presented in Supplementary table 4. We finally included 14 articles corresponding to 20 studies whose characteristics are presented in the Supplementary table 5 [39–52]. The majority of included studies enrolled participants less than one year (9 studies) who did not receive prophylaxis against HRSV (9 studies), and were conducted in the United States (10 studies). The included studies were published between 1990 and 2018. Study participants were recruited between 1985 and 2017.

Figure 1: Flow chart for the systematic literature search

Included studies risk of bias assessment

The majority of studies had a low risk of bias (18 studies, Supplemental table 6). Most of the included studies were population-based (15 studies) and therefore representative of the population source. All FT children were recruited from the same population as the PT infants and therefore had no selection bias. Gestational age data were collected from secure patient file and HRSV infections were confirmed by laboratory assays, ICD codes or secure recordings in almost all included studies (18 studies). The majority of included studies did not match participants on age at inclusion (17 studies). Some studies, however, excluded participants with comorbidities, thereby reducing the confounding factors of these parameters on the effect of prematurity on health care utilization reported by the study.

Meta-analyses and sensitivity analyses

Fifteen studies on the association between premature birth and ICU admission shown a combined RR of 2.6 (95% CI = 1.9-3.5, I2 = 83.2%) (Figure 2). The CFR was significantly higher in PT infants enrolled in six studies (RR = 6.9, 95% CI = 2.0-23.8, I2 = 5.2%). Children born prematurely had LOS at hospital (SMD = 0.6, 95% CI = 0.5; 0.8, I2 = 77.8%) and at ICU (SMD = 0.6, 95% CI = 0.4; 0.8, I2 = 75.0%) significantly higher than FT infants (Figure 3). There was no difference for age at the time of the first episode of HRSV infection between PT and FT infants (SMD = 0.0, 95% CI = -0.0; 0.1, I2 = 16.6%). The frequency of ED visits (RR = 1.1, 95% CI = 0.5-2.1, I2 = 62.6%) and oxygen supplementation (RR = 1.2, 95% CI = 0.9-1.6, I2 = 91.4%) were not different between PT and FT infants. There was no association in the overall analysis of the risk of MV utilization between PT and FT infants (RR = 1.9, 95% CI = 0.5-7.1, I2 = 94.8). %), but sensitivity analysis with only children aged ≤ 2 years shows a significantly higher risk in PT infants (RR = 15.5, 95% CI = 8.9-26.9, I2 =

0.0%) (Tables 1 and 2). Results of sensitivity analyzes that included only low-risk studies and children ≤ 2 years of age did not affect RR or 95% CI for ICU admission, ED visits, oxygen supplementation, CFR, LOS at hospital and at ICU, and age at time of index HRSV infection, suggesting the robustness of our meta-analyses.

Figure 2. Forest plot for the association between premature birth and risk of health care use for binary outcomes.

Figure 3. Forest plot for the association between premature birth and the risk of health care use for continuous outcomes.

Table 1: Meta-analyses and sensitivity analyses of binary outcomes of health care resource utilization among preterm and term infants hospitalized with HRSV infections

	RR (95%CI)	95% Prediction interval	N Studies	N Preterm infants	N Term infants	H¶ (95%CI)	I^{2}§ (95%CI)	P-value heterogeneity	P-value Egger test
**Intensive care unit admission**									
**Overall**	2.6 [1.9 - 3.5]	[0.8 - 8.1]	15	1640	12884	2.4 [1.9 - 3.1]	83.2 [73.6 - 89.3]	< 0.001	0.275
**≤ 2 years**	2.3 [1.6 - 3.2]	[0.7 - 7.3]	11	1316	10846	2.7 [2.1 - 3.5]	86.5 [77.8 - 91.9]	< 0.001	0.141
**Low risk of bias**	2.7 [2.0 - 3.7]	[0.8 - 8.5]	14	1627	12830	2.5 [2.0 - 3.2]	84.1 [74.7 – 90.0]	< 0.001	0.349
**Emergency department visits**									
**Overall**	1.1 [0.9 - 1.2]	[0.4 - 2.7]	3	89	645	1.5 [1.0 - 2.8]	54 [0.0 - 86.8]	0.114	0.027
**≤ 2 years**	1.1 [0.9 - 1.2]	[0.4 - 2.7]	3	89	645	1.5 [1.0 - 2.8]	54 [0.0 - 86.8]	0.114	0.027
**Low risk of bias**	1.1 [0.9 - 1.2]	[0.4 - 2.7]	3	89	645	1.5 [1.0 - 2.8]	54 [0.0 - 86.8]	0.114	0.027
**Mechanical ventilation utilization**									
**Overall**	1.9 [0.5 - 7.1]	[0.0 - 177.4]	6	793	5054	4.4 [3.4 - 5.7]	94.8 [91.1 - 96.9]	< 0.001	0.652
**≤ 2 years**	15.5 [8.9 - 26.9]	NA	2	571	4596	1.0	0.0	0.656	NA
**Low risk of bias**	2.1 [0.4 - 10.7]	[0.0 - 788.3]	5	753	4952	3.5 [2.5 - 4.9]	91.7 [83.5 - 95.8]	< 0.001	0.229
**Supplemental oxygen utilization**									
**Overall**	1.2 [0.9 - 1.6]	[0.4 - 4]	5	461	1444	3.4 [2.4 - 4.8]	91.4 [83.0 - 95.7]	< 0.001	0.424
**≤ 2 years**	1.2 [0.9 - 1.7]	NA	2	279	1088	5.1 [3.0 - 8.5]	96.1 [89.0 - 98.6]	< 0.001	NA
**Low risk of bias**	1.3 [0.8 - 1.9]	[0.2 - 7.8]	4	448	1390	2.9 [1.9 - 4.4]	87.7 [70.9 - 94.8]	< 0.001	0.435
**Case fatality rate**									

	RR (95%CI)	95% Prediction interval	N Studies	N Preterm infants	N Term infants	H[¶] (95%CI)	I^{2}[§] (95%CI)	P-value heterogeneity	P-value Egger test
**Overall**	6.9 [2.0 - 23.8]	NA	2	709	3277	1.0	5.2	0.304	NA
**≤ 2 years**	6.9 [2.0 - 23.8]	NA	2	514	1901	1.0	5.2	0.304	NA
**Low risk of bias**	6.9 [2.0 - 23.8]	NA	2	669	3175	1.0	5.2	0.304	NA

RR: Risk Ratio; N: Number; 95% CI: 95% Confidence Interval; NA: Not Applicable; LOS: Length of stay;

¶H is a measure of the extent of heterogeneity, a value of H =1 indicates homogeneity of effects and a value of H >1indicates a potential heterogeneity of effects.

§: I2 describes the proportion of total variation in study estimates that is due to heterogeneity, a value > 50% indicates presence of heterogeneity

Table 2: Meta-analyses and sensitivity analyses of continuous outcomes of health care resource utilization among preterm and term infants hospitalized with HRSV infections

	SMD (95%CI)	95% Prediction interval	N Studies	N Preterm infants	N Term infants	H[¶] (95%CI)	$I^{2\S}$ (95%CI)	P-value heterogeneity	P-value Egger test
**Hospitalization LOS**									
**Overall**	0.6 [0.5 ; 0.8]	[0.1 ; 1.1]	12	1441	19945	2.1 [1.6 - 2.8]	77.8 [61.6 - 87.2]	< 0.001	0,763
**Low risk of bias**	0.6 [0.5 ; 0.8]	[0.1 ; 1.1]	12	1441	19945	2.1 [1.6 - 2.8]	77.8 [61.6 - 87.2]	< 0.001	0,763
**≤ 2 years**	0.7 [0.5 ; 0.9]	[0.0 ; 1.4]	7	616	5681	2.1 [1.4 - 3]	77.1 [52.1 - 89]	< 0.001	0,739
**Intensive care unit LOS**									
**Overall**	0.6 [0.4 ; 0.8]	[-0.1 ; 1.2]	7	608	5747	2 [1.4 - 2.9]	75.0 [47.0 - 88.2]	0,001	0,262
**Low risk of bias**	0.6 [0.3 ; 0.8]	[-0.2 ; 1.3]	6	568	5645	2.2 [1.5 - 3.2]	78.8 [53.5 - 90.3]	< 0.001	0,333
**≤ 2 years**	0.6 [0.3 ; 0.8]	[-0.2 ; 1.3]	6	568	5645	2.2 [1.5 - 3.2]	78.8 [53.5 - 90.3]	< 0.001	0,333
**Age at time of index HRSV infection**									
**Overall**	0.0 [-0.0 ; 0.1]	[-0.1 ; 0.2]	5	904	17910	1.1 [1 - 2.4]	16.6 [0.0 - 82.6]	0,309	0,622
**Low risk of bias**	0.0 [-0.0 ; 0.1]	[-0.1 ; 0.2]	5	904	17910	1.1 [1 - 2.4]	16.6 [0.0 - 82.6]	0,309	0,622
**≤ 2 years**	0.1 [-0.1 ; 0.3]	[-1.9 ; 2.2]	3	261	4002	1.3 [1 - 2.4]	41.5 [0.0 - 82.2]	0,181	0,820

SMD: Standardised Mean Difference; N: Number; 95% CI: 95% Confidence Interval; NA: Not Applicable; LOS: Length of stay;

¶H is a measure of the extent of heterogeneity, a value of H =1 indicates homogeneity of effects and a value of H >1indicates a potential heterogeneity of effects.

§: I2 describes the proportion of total variation in study estimates that is due to heterogeneity, a value > 50% indicates presence of heterogeneity

Heterogeneity analyses and publication Bias

The studies included in the analyses of ED visits (I2 = 54.0%, P = 0.114), CFR (I2 = 5.2%, P = 0.304), and age at the first episode of HRSV infection (I2 = 16.6%, P = 0.309) were homogeneous. Significant heterogeneity was observed in the remaining analyses. The funnel plot was asymmetric for studies involving the ED visits (P Egger = 0.027, Supplemental figure 2). No publication bias was recorded for the remaining analyses (Supplemental figures 1-10).

Subgroup Analysis

The risk of admission to ICU was significantly higher in PT compared to FT in all WHO regions represented in the review (Supplementary tables 7 and 8). An increased rate of ICU admission in PT infants was found in the Western Pacific (RR = 5.9, 95% CI = 4.6-7.5) and Europe (RR = 3.5, 95% CI = 2.9-4.2) compared to America (RR = 1.8, 95% CI = 1.4-2.2) ($p < 0.001$). The use of MV was significantly greater in PT children who did not receive prophylaxis against HRSV ($p = 0.002$). The risk of MV utilization in PT infants was significantly higher ($p <0.001$) in the Western Pacific (RR = 15.9, 95% CI = 9.1-27.9), in America (RR = 5.2, CI 95% = 2.4-11.7) compared to Europe (RR = 0.6, 95% CI = 0.3-1.4) where there was no association. The use of MV was statistically higher among PT compared to FT infants in the studies that identified HRSV from International Classification of Disease codes (RR = 15.5, 95% CI = 8.9-26.9) compared to the authors who reported the use of laboratory assays for HRSV identification (RR = 0.3, 95% CI = 0.1-0.8) ($p = 0.007$). There was a significant difference in the subgroup analysis according to age at the time of the first episode of HRSV infection by gestational age of PT children ($p = 0.047$). Children born at a gestational age ≤ 32 wGA had an age at the time of the first episode of HRSV infection

greater than FT infants (SMD = 0.4, 95% CI = 0.0, 0.7). There was no difference in the remaining subgroup analyses by gestational age of PT infants, prophylaxis against HRSV, HRSV detection assays, and WHO region.

Confounding factors

The majority of studies that provided primary data on confounding factors had a symmetric distribution between PT and FT infants (Supplemental tables 9) including, HRSV/other virus codetection (2/2), age ≤ 3 months before/during the HRSV season (1/1), history of asthma in the family (1/1), bacterial codetection (1/1), breastfeeding < 2 months (1/1), daycare attendance during the first year (1/1), the presence of siblings of more than one year (1/1), smoking during pregnancy (1/1), twins (1/1), male gender (7/11), and bronchopulmonary dysplasia (3/4). Half of the studies with data on heart disease (3/6) and respiratory co-morbidities (1/2) were equally distributed between PT and FT infants. All studies (4/4) with data on at least one underlying medical condition and passive smoking during the first year of life (1/1) had an inequitable distribution between PT and FT infants. The sensitivity analyses with symmetric distribution of each confounding factors examined revealed no major influence on the overall estimated results (supplementary tables 10 and 11). The increased frequency of ICU admission was lost (RR = 1.2, 95% CI = 0.3-5.1) in a study with a symmetric distribution of children with bronchopneumonia dysplasia [52]. The increased hospital LOS in PT infants was lost in a study with symmetric distribution of day-care attendance 1st year (SMD = 0.0, 95% CI = -0.8; 0.8) and bronchopneumonia dysplasia (SMD = 0.4, 95% CI = -0.5; 1.3) [43].

Discussion

This study showed that compared to FT, PT infants hospitalized for HRSV infections had a greater rate of ICU admission. Premature infants hospitalized for HRSV infections also had longer stays in the hospital and ICU than FT infants. The CFR was also higher in PT hospitalized for HRSV infections compared to FT infants. No significant differences in ED visits, age at first HRSV infection, oxygen utilization, and use of MV were observed between PT and FT infants hospitalized for HRSV infections. The use of MV, however, was significantly more common in PT infants under 2 years hospitalized for HRSV infections. Preterm infants < 32 wGA experienced the first episode of HRSV infection later than FT infants. Admission to the ICU and use of MV was significantly more common in a study of the western Pacific (Taiwan) compared to Europe and America. The risk of ICU stay was more prevalent in Europe compared to America while the risk of MV was more prevalent in America compared to Europe. Finally, we found that prophylaxis against HRSV reduced the risk of MV in the included studies.

The results obtained from this systematic review and meta-analysis are consistent with that of a previous systematic review conducted without meta-analysis with respect to increased frequency in PT compared to FT infants for admission to ICU, LOS in hospital and ICU, and CFR. One explanation for this difference between PT and FT infants may be incomplete pulmonary and immature development of PT infants, which makes them more vulnerable to death and more severe HRSV infections, resulting in longer rates and hospital stays [53–58]. The systematic review conducted by Figueras-Aloy et al. reported that there was no difference in the use of MV and LOS in hospital between PT and FT infants. In the present study,

however, a sensitivity analysis in patients aged ≤ 2 years revealed a high frequency of MV use in PT compared to FT infants. Contrary to the present systematic review which shows no difference in the frequency of oxygen supplementation in PT than in FT infants, the Figueras-Aloy et al. had reported controversial results in this regard [15]. This review highlights for the first time a similar rate of ED visit between PT and FT children. We had also shown in this review that there is no difference in age at the first episode of HRSV between PT and FT infants. This result is consistent with the review conducted by Parikh et al. on US published data and databases on patient hospitalized for HRSV infections from 2006 to 2011 [59]. While term infants are protected from HRSV infections during the first months of life by maternal antibodies [60], children born at ≤ 32 wGA are also protected by their prolonged stay in hospitals at birth [61]. This hypothesis is well supported by the subgroup analysis conducted in this work which revealed that children of ≤ 32 wGA were infected with HRSV later than FT infants. The management of HRSV infections varies considerably in countries according to economic and socio-cultural criteria, which greatly compromises the possibility of interpreting differences in the use of hospital care resources according to the WHO regions observed during this work. Several studies have shown that prophylaxis with Palivizumab helps reduce the risk of hospitalization and LOS in hospital in PT children [21,62,63]. This work has shown that children who received palivizumab did not have a greater risk of using MV.

This is the first meta-analysis to establish the association between gestational age and the use of health care resources in patients with HRSV infection. Our results were controlled for multiple confounding factors including breastfeeding, bronchopulmonary dysplasia, daycare attendance, sex, age before / during the HRSV season, tobacco exposure, family antecedent of asthma, underlying medical conditions, viral and bacterial codetections, overcrowding and

multiple gestations. Apart from a study conducted in the Western Pacific west, the remaining originated from America and Europe, this represents on the one hand a weakness for this review by compromising the generalizability of findings globally and is also a strength by highlighting a gap of knowledge for other regions of the world consisting mainly of countries with limited resources that could have a particular profile and have a strong specificity in relation to the results of this work. Difference between PT with co-morbid conditions and otherwise healthy PT is also important for HRSV prophylaxis administration guidelines. The included studies in this review did not report the frequency of health care use stratified for healthy and PT with comorbidities. We were therefore not able to do this analysis as part of this review. Some included studies (5/20) used ICD codes for HRSV identification. These records are likely to be affected by inaccurate coding and may therefore incorrectly lead to the inclusion of negative HRSV participants. With the exception of the use of MV category, the group analyzes conducted during this work did not reveal any difference between these studies with the ICD codes and the studies specifying the laboratory assay used. The small number of studies included in this review is also one of its limitations. Multiple categories of subgroup analyzes were represented by a single category or were not possible due to lack of data. Another limitation of this work is the retrospective nature of the included studies that may be affected by multiple biases, including selection bias, recall bias, and the difficulty of verifying the data collected in the archives by authors of included studies.

The results of this systematic review indicate that compared to FT hospitalized for HRSV infections, those with a history of premature birth are at increased risk of admission to ICU, leading to longer hospitalizations LOS, a higher rate of MV utilization, and higher mortality. Our results also indicate no difference between age at first episode of HRSV infection, frequency of ED visits, and oxygen supplemental utilization between PT and FT

infants. In addition, according to the results of this work, compared to FT infants, there is no difference in the use of health care resources between early (< 32/34 wGA) and late PT infants.

Pediatricians and stakeholder[illegible]ould be aware that compared to FT children, early and late PT hospitalized for HRSV infections are at high risk for increased use of medical resources and poor outcomes. Improving strategies for administering HRSV prophylaxis and finalizing the development of the HRSV vaccine, especially vaccine dedicated to pregnant women [64], could help reduce the use of health care resources and the mortality caused by HRSV infections in PT infants. Resource-limited areas such as Africa and Southeast Asia have virtually no access to or policies for the administration of HRSV prophylaxis and could thus have a particular profile for the epidemiology of HRSV infections. Additional research from these resource-limited areas is therefore important to fully understand the relationship between gestational age and HRSV infections at the global level. Additional studies of the evaluation of interventions to reduce the use of care resources by HRSV-infected premature infants are also needed.

Acknowledgment

None

References

1. Hall CB, Weinberg GA, Iwane MK, Blumkin AK, Edwards KM, Staat MA, Auinger P, Griffin MR, Poehling KA, Erdman D, Grijalva CG, Zhu Y, Szilagyi P. The Burden of Respiratory Syncytial Virus Infection in Young Children. New England Journal of Medicine. 2009 Feb 5;360(6):588–98.

2. Nair H, Nokes DJ, Gessner BD, Dherani M, Madhi SA, Singleton RJ, O'Brien KL, Roca A, Wright PF, Bruce N, Chandran A, Theodoratou E, Sutanto A, Sedyaningsih ER, Ngama M, Munywoki PK, Kartasasmita C, Simões EA, Rudan I, Weber MW, Campbell H. Global burden of acute lower respiratory infections due to respiratory syncytial virus in young children: a systematic review and meta-analysis. Lancet. 2010 May 1;375(9725):1545–55.

3. Shi T, McAllister DA, O'Brien KL, Simoes EAF, Madhi SA, Gessner BD, Polack FP, Balsells E, Acacio S, Aguayo C, Alassani I, Ali A, Antonio M, Awasthi S, Awori JO, Azziz-Baumgartner E, Baggett HC, Baillie VL, Balmaseda A, Barahona A, Basnet S, Bassat Q, Basualdo W, Bigogo G, Bont L, Breiman RF, Brooks WA, Broor S, Bruce N, Bruden D, Buchy P, Campbell S, Carosone-Link P, Chadha M, Chipeta J, Chou M, Clara W, Cohen C, de Cuellar E, Dang D-A, Dash-Yandag B, Deloria-Knoll M, Dherani M, Eap T, Ebruke BE, Echavarria M, de Freitas Lázaro Emediato CC, Fasce RA, Feikin DR, Feng L, Gentile A, Gordon A, Goswami D, Goyet S, Groome M, Halasa N, Hirve S, Homaira N, Howie SRC, Jara J, Jroundi I, Kartasasmita CB, Khuri-Bulos N, Kotloff KL, Krishnan A, Libster R, Lopez O, Lucero MG, Lucion F, Lupisan SP, Marcone DN, McCracken JP, Mejia M, Moisi JC, Montgomery JM, Moore DP, Moraleda C, Moyes J, Munywoki P, Mutyara K, Nicol MP, Nokes DJ, Nymadawa P, da Costa Oliveira MT,

Oshitani H, Pandey N, Paranhos-Baccalà G, Phillips LN, Picot VS, Rahman M, Rakoto-Andrianarivelo M, Rasmussen ZA, Rath BA, Robinson A, Romero C, Russomando G, Salimi V, Sawatwong P, Scheltema N, Schweiger B, Scott JAG, Seidenberg P, Shen K, Singleton R, Sotomayor V, Strand TA, Sutanto A, Sylla M, Tapia MD, Thamthitiwat S, Thomas ED, Tokarz R, Turner C, Venter M, Waicharoen S, Wang J, Watthanaworawit W, Yoshida L-M, Yu H, Zar HJ, Campbell H, Nair H, RSV Global Epidemiology Network. Global, regional, and national disease burden estimates of acute lower respiratory infections due to respiratory syncytial virus in young children in 2015: a systematic review and modelling study. Lancet. 2017 Jul 6;

4. Glezen WP, Taber LH, Frank AL, Kasel JA. Risk of primary infection and reinfection with respiratory syncytial virus. Am J Dis Child. 1986 Jun;140(6):543–6.

5. Makari D, Staat MA, Henrickson KJ, Wu X, Ambrose CS. The Underrecognized Burden of Respiratory Syncytial Virus Among Infants Presenting to US Emergency Departments. Clin Pediatr (Phila). 2015 Jun;54(6):594–7.

6. Reeves RM, Hardelid P, Gilbert R, Warburton F, Ellis J, Pebody RG. Estimating the burden of respiratory syncytial virus (RSV) on respiratory hospital admissions in children less than five years of age in England, 2007-2012. Influenza Other Respir Viruses. 2017 Jan 6;

7. El Kholy AA, Mostafa NA, El-Sherbini SA, Ali AA, Ismail RI, Magdy RI, Hamdy MS, Soliman MS. Morbidity and outcome of severe respiratory syncytial virus infection: Outcome of severe RSV infection. Pediatrics International. 2013 Jun;55(3):283–8.

8. Geoghegan S, Erviti A, Caballero MT, Vallone F, Zanone SM, Losada JV, Bianchi A, Acosta PL, Talarico LB, Ferretti A, Grimaldi LA, Sancilio A, Dueñas K, Sastre G,

Rodriguez A, Ferrero F, Barboza E, Gago GF, Nocito C, Flamenco E, Perez AR, Rebec B, Ferolla FM, Libster R, Karron RA, Bergel E, Polack FP. Mortality due to Respiratory Syncytial Virus: Burden and Risk Factors. Am J Respir Crit Care Med [Internet]. 2016 Jun 22 [cited 2016 Jun 28]; Available from: http://hinarilogin.research4life.org/uniquesigwww.atsjournals.org/uniquesig0/doi/abs/10.1164/rccm.201603-0658OC

9. Okiro EA, Ngama M, Bett A, Nokes DJ. The Incidence and Clinical Burden of Respiratory Syncytial Virus Disease Identified through Hospital Outpatient Presentations in Kenyan Children. Cowling BJ, editor. PLoS ONE. 2012 Dec 26;7(12):e52520.

10. Bhuiyan MU, Luby SP, Alamgir NI, Homaira N, Sturm-Ramirez K, Gurley ES, Abedin J, Zaman RU, Alamgir A, Rahman M, Ortega-Sanchez IR, Azziz-Baumgartner E. Costs of hospitalization with respiratory syncytial virus illness among children aged <5 years and the financial impact on households in Bangladesh, 2010. J Glob Health. 2017 Jun;7(1):010412.

11. Blake SM, Tanaka D, Bendz LM, Staebler S, Brandon D. Evaluation of the Financial and Health Burden of Infants at Risk for Respiratory Syncytial Virus. Adv Neonatal Care. 2016 Dec 6;

12. Heikkinen T, Ojala E, Waris M. Clinical and Socioeconomic Burden of Respiratory Syncytial Virus Infection in Children. J Infect Dis. 2016 Oct 12;

13. Bont L, Checchia PA, Fauroux B, Figueras-Aloy J, Manzoni P, Paes B, Simoes EA, Carbonell-Estrany X. Defining the Epidemiology and Burden of Severe Respiratory

Syncytial Virus Infection Among Infants and Children in Western Countries. Infectious diseases and therapy. 2016 Sep;5:271–98.

14. Shi T, Balsells E, Wastnedge E, Singleton R, Rasmussen ZA, Zar HJ, Rath BA, Madhi SA, Campbell S, Vaccari LC, Bulkow LR, Thomas ED, Barnett W, Hoppe C, Campbell H, Nair H. Risk factors for respiratory syncytial virus associated with acute lower respiratory infection in children under five years: Systematic review and meta–analysis. J Glob Health [Internet]. 2015 Dec [cited 2015 Dec 22];5(2). Available from: http://www.ncbi.nlm.nih.gov/pmc/articles/PMC4676580/

15. Figueras-Aloy J, Manzoni P, Paes B, Simões EAF, Bont L, Checchia PA, Fauroux B, Carbonell-Estrany X. Defining the Risk and Associated Morbidity and Mortality of Severe Respiratory Syncytial Virus Infection Among Preterm Infants Without Chronic Lung Disease or Congenital Heart Disease. Infect Dis Ther. 2016 Sep 14;

16. Giersing BK, Karron RA, Vekemans J, Kaslow DC, Moorthy VS. Meeting report: WHO consultation on Respiratory Syncytial Virus (RSV) vaccine development, Geneva, 25-26 April 2016. Vaccine. 2017 Mar 14;

17. Caldwell NA, Townsend C. Where are we with RSV prophylaxis? Arch Dis Child Educ Pract Ed. 2016 Feb;101(1):38–42.

18. Rivera CA, Gómez RS, Díaz RA, Céspedes PF, Espinoza JA, González PA, Riedel CA, Bueno SM, Kalergis AM. Novel therapies and vaccines against the human respiratory syncytial virus. Expert Opinion on Investigational Drugs. 2015 Oct 12;0(0):1–18.

19. Xing Y, Proesmans M. New therapies for acute RSV infections: where are we? Eur J Pediatr. 2019 Feb;178(2):131–8.

20. Zembles TN, Gaertner KM, Gutzeit MF, Willoughby RE. Implementation of American Academy of Pediatrics guidelines for palivizumab prophylaxis in a pediatric hospital. Am J Health Syst Pharm. 2016 Mar 15;73(6):405–8.

21. The IMpact-RSV Study Group. Palivizumab, a humanized respiratory syncytial virus monoclonal antibody, reduces hospitalization from respiratory syncytial virus infection in high-risk infants. The IMpact-RSV Study Group. Pediatrics. 1998 Sep;102(3 Pt 1):531–7.

22. American Academy of Pediatrics Committee on Infectious Diseases, American Academy of Pediatrics Bronchiolitis Guidelines Committee. Updated guidance for palivizumab prophylaxis among infants and young children at increased risk of hospitalization for respiratory syncytial virus infection. Pediatrics. 2014 Aug;134(2):415–20.

23. Rajah B, Sánchez PJ, Garcia-Maurino C, Leber A, Ramilo O, Mejias A. Impact of the Updated Guidance for Palivizumab Prophylaxis against Respiratory Syncytial Virus Infection: A Single Center Experience. J Pediatr. 2016 Nov 15;

24. Kong AM, Krilov LR, Fergie J, Goldstein M, Diakun D, Wade SW, Pavilack M, McLaurin KK. The 2014–2015 National Impact of the 2014 American Academy of Pediatrics Guidance for Respiratory Syncytial Virus Immunoprophylaxis on Preterm Infants Born in the United States. Am J Perinatol. 2018 Jan;35(2):192–200.

25. Grindeland CJ, Mauriello CT, Leedahl DD, Richter LM, Meyer AC. Association Between Updated Guideline-Based Palivizumab Administration and Hospitalizations for Respiratory Syncytial Virus Infections. Pediatr Infect Dis J. 2016;35(7):728–32.

26. Zembles TN, Bushee GM, Willoughby RE. Impact of American Academy of Pediatrics Palivizumab Guidance for Children ≥29 and <35 Weeks of Gestational Age. J Pediatr. 2019 Jun;209:125–9.

27. Krilov LR, Fergie J, Goldstein M, Brannman L. Impact of the 2014 American Academy of Pediatrics Immunoprophylaxis Policy on the Rate, Severity, and Cost of Respiratory Syncytial Virus Hospitalizations among Preterm Infants. Am J Perinatol. 2019 Aug 20;

28. Coffman S. Late preterm infants and risk for RSV. MCN Am J Matern Child Nurs. 2009 Dec;34(6):378–84; quiz 385–6.

29. Moher D, Liberati A, Tetzlaff J, Altman DG. Preferred Reporting Items for Systematic Reviews and Meta-Analyses: The PRISMA Statement. PLoS Med [Internet]. 2009 Jul 21 [cited 2018 Sep 11];6(7). Available from: https://www.ncbi.nlm.nih.gov/pmc/articles/PMC2707599/

30. Engle WA. A recommendation for the definition of “late preterm” (near-term) and the birth weight-gestational age classification system. Semin Perinatol. 2006 Feb;30(1):2–7.

31. Wells G, Shea B, O’Connell D, Peterson J, Welch V, Losos M, Tugwell P. The Newcastle-Ottawa Scale (NOS) for assessing the quality of nonrandomised studies in meta-analyses [Internet]. 2000 [cited 2017 Sep 25]. Available from: http://www.ohri.ca/programs/clinical_epidemiology/oxford.asp

32. Schwarzer G. meta: An R package for meta-analysis. R News. 2007;7(3):40–5.

33. R Core Team. R: A language and environment for statistical computing. R Foundation for Statistical Computing, Vienna, Austria. [Internet]. 2017 [cited 2018 Sep 28]. Available from: https://www.r-project.org/

34. Higgins JPT, Thompson SG. Quantifying heterogeneity in a meta-analysis. Stat Med. 2002 Jun 15;21(11):1539–58.

35. O’Connor AM. Interpretation of odds and risk ratios. J Vet Intern Med. 2013 Jun;27(3):600–3.

36. Takeshima N, Sozu T, Tajika A, Ogawa Y, Hayasaka Y, Furukawa TA. Which is more generalizable, powerful and interpretable in meta-analyses, mean difference or standardized mean difference? BMC Med Res Methodol. 2014 Feb 21;14:30.

37. Wan X, Wang W, Liu J, Tong T. Estimating the sample mean and standard deviation from the sample size, median, range and/or interquartile range. BMC Med Res Methodol [Internet]. 2014 Dec 19 [cited 2019 Feb 1];14. Available from: https://www.ncbi.nlm.nih.gov/pmc/articles/PMC4383202/

38. Egger M, Davey Smith G, Schneider M, Minder C. Bias in meta-analysis detected by a simple, graphical test. BMJ. 1997 Sep 13;315(7109):629–34.

39. Assefa D, Amin N, Dozor AJ, Parton LA. Attenuated interleukin-8/leukocyte immunoresponse in preterm infants compared with term infants hospitalized with respiratory syncytial virus bronchiolitis: a pilot study. Hum Immunol. 2011 Sep;72(9):708–11.

40. Chi H, Chang I-S, Tsai F-Y, Huang L-M, Shao P-L, Chiu N-C, Chang L-Y, Huang F-Y. Epidemiological study of hospitalization associated with respiratory syncytial virus infection in Taiwanese children between 2004 and 2007. J Formos Med Assoc. 2011 Jun;110(6):388–96.

41. Followell S, Christopherson M, Davis P. Late preterm infants at greater risk of paediatric intensive care admission and longer invasive ventilation due to respiratory syncitial virus bronchiolitis. Archives of Disease in Childhood. 2018 Mar 1;103(Suppl 1):A13–A13.

42. Forbes ML, Hall CB, Jackson A, Masaquel AS, Mahadevia PJ. Comparative costs of hospitalisation among infants at high risk for respiratory syncytial virus lower respiratory tract infection during the first year of life. J Med Econ. 2010 Mar;13(1):136–41.

43. Gijtenbeek RGP, Kerstjens JM, Reijneveld SA, Duiverman EJ, Bos AF, Vrijlandt EJLE. RSV infection among children born moderately preterm in a community-based cohort. European Journal of Pediatrics. 2015 Apr;174(4):435–42.

44. Greenberg D, Dagan R, Shany E, Bar-Ziv J, Givon-Lavi N. Increased risk for respiratory syncytial virus-associated, community-acquired alveolar pneumonia in infants born at 31-36 weeks of gestation. Pediatr Infect Dis J. 2014 Apr;33(4):381–6.

45. Gross I, Siedner-Weintraub Y, Abu Ahmad W, Bar-Oz B, Eventov-Friedman S. National Evidence in Israel Supporting Reevaluation of Respiratory Syncytial Virus Prophylactic Guidelines. Neonatology. 2017;111(3):240–6.

46. Helfrich AM, Nylund CM, Eberly MD, Eide MB, Stagliano DR. Healthy Late-preterm infants born 33-36+6 weeks gestational age have higher risk for respiratory syncytial virus hospitalization. Early Hum Dev. 2015 Sep;91(9):541–6.

47. Horn SD, Smout RJ. Effect of prematurity on respiratory syncytial virus hospital resource use and outcomes. J Pediatr. 2003 Nov;143(5 Suppl):S133-141.

48. Leader S, Yang H, DeVincenzo J, Jacobson P, Marcin JP, Murray DL. Time and Out-of-Pocket Costs Associated with Respiratory Syncytial Virus Hospitalization of Infants. Value in Health. 2003 Mar 1;6(2):100–6.

49. Meert K, Heidemann S, Abella B, Sarnaik A. Does prematurity alter the course of respiratory syncytial virus infection? Crit Care Med. 1990 Dec;18(12):1357–9.

50. Resch B, Gusenleitner W, Mueller WD. Risk of concurrent bacterial infection in preterm infants hospitalized due to respiratory syncytial virus infection. Acta Paediatr. 2007 Apr;96(4):495–8.

51. Van de Steen O, Miri F, Gunjaca M, Klepac V, Gross B, Notario G, Wegzyn CM. The Burden of Severe Respiratory Syncytial Virus Disease Among Children Younger than 1 Year in Central and Eastern Europe. Infect Dis Ther. 2016 Jun;5(2):125–37.

52. Vo P, Mitchell I, Gooch KL, Manthena S, Holton MJ, Ruff M. The Disease Burden Associated With Infant Hospitalization For A Lower Respiratory Tract Infection (Lrti) In Canada. Value in Health. 2014 May 1;17(3):A179.

53. Ochola R, Sande C, Fegan G, Scott PD, Medley GF, Cane PA, Nokes DJ. The Level and Duration of RSV-Specific Maternal IgG in Infants in Kilifi Kenya. PLoS One [Internet]. 2009 Dec 2 [cited 2015 Nov 13];4(12). Available from: http://www.ncbi.nlm.nih.gov/pmc/articles/PMC2779853/

54. Borchers AT, Chang C, Gershwin ME, Gershwin LJ. Respiratory Syncytial Virus—A Comprehensive Review. Clinic Rev Allerg Immunol. 2013 Apr 12;45(3):331–79.

55. Colin AA, McEvoy C, Castile RG. Respiratory Morbidity and Lung Function in Preterm Infants of 32 to 36 Weeks' Gestational Age. Pediatrics. 2010 Jul;126(1):115–28.

56. Friedrich L, Pitrez PMC, Stein RT, Goldani M, Tepper R, Jones MH. Growth rate of lung function in healthy preterm infants. Am J Respir Crit Care Med. 2007 Dec 15;176(12):1269–73.

57. Maritz GS, Morley CJ, Harding R. Early developmental origins of impaired lung structure and function. Early Hum Dev. 2005 Sep;81(9):763–71.

58. Langston C, Kida K, Reed M, Thurlbeck WM. Human lung growth in late gestation and in the neonate. Am Rev Respir Dis. 1984 Apr;129(4):607–13.

59. Parikh RC, McLaurin KK, Margulis AV, Mauskopf J, Ambrose CS, Pavilack M, Candrilli SD. Chronologic Age at Hospitalization for Respiratory Syncytial Virus Among Preterm and Term Infants in the United States. Infect Dis Ther. 2017 Dec;6(4):477–86.

60. Glezen WP, Paredes A, Allison JE, Taber LH, Frank AL. Risk of respiratory syncytial virus infection for infants from low-income families in relationship to age, sex, ethnic group, and maternal antibody level. J Pediatr. 1981 May;98(5):708–15.

61. La Gamma EF, Kumar VR, Wadhawan R, Ye S, Sifakis F, Ycas J, Ambrose CS. Receipt of palivizumab before birth hospitalization discharge among preterm infants in the United States. Am J Perinatol. 2015 Sep;32(11):1017–23.

62. Carbonell-Estrany X, Fullarton JR, Gooch KL, Vo PG, Figueras-Aloy J, Lanari M, Gouyon JB, Liese JG. Effects of parental and household smoking on the risk of respiratory syncytial virus (RSV) hospitalisation in late-preterm infants and the potential impact of RSV prophylaxis. The journal of maternal-fetal & neonatal medicine : the official journal of the European Association of Perinatal Medicine, the Federation of

Asia and Oceania Perinatal Societies, the International Society of Perinatal Obstet. 2013 Jun;26:926–31.

63. Doering G, Gusenleitner W, Belohradsky BH, Burdach S, Resch B, Liese JG. The risk of respiratory syncytial virus-related hospitalizations in preterm infants of 29 to 35 weeks' gestational age. Pediatr Infect Dis J. 2006 Dec;25(12):1188–90.

64. Beugeling M, De Zee J, Woerdenbag HJ, Frijlink HW, Wilschut JC, Hinrichs WLJ. Respiratory syncytial virus subunit vaccines based on the viral envelope glycoproteins intended for pregnant women and the elderly. Expert Rev Vaccines. 2019 Aug 25;1–16.

Figure

Click here to download Figure Figure 1_Flow Diagram.docx

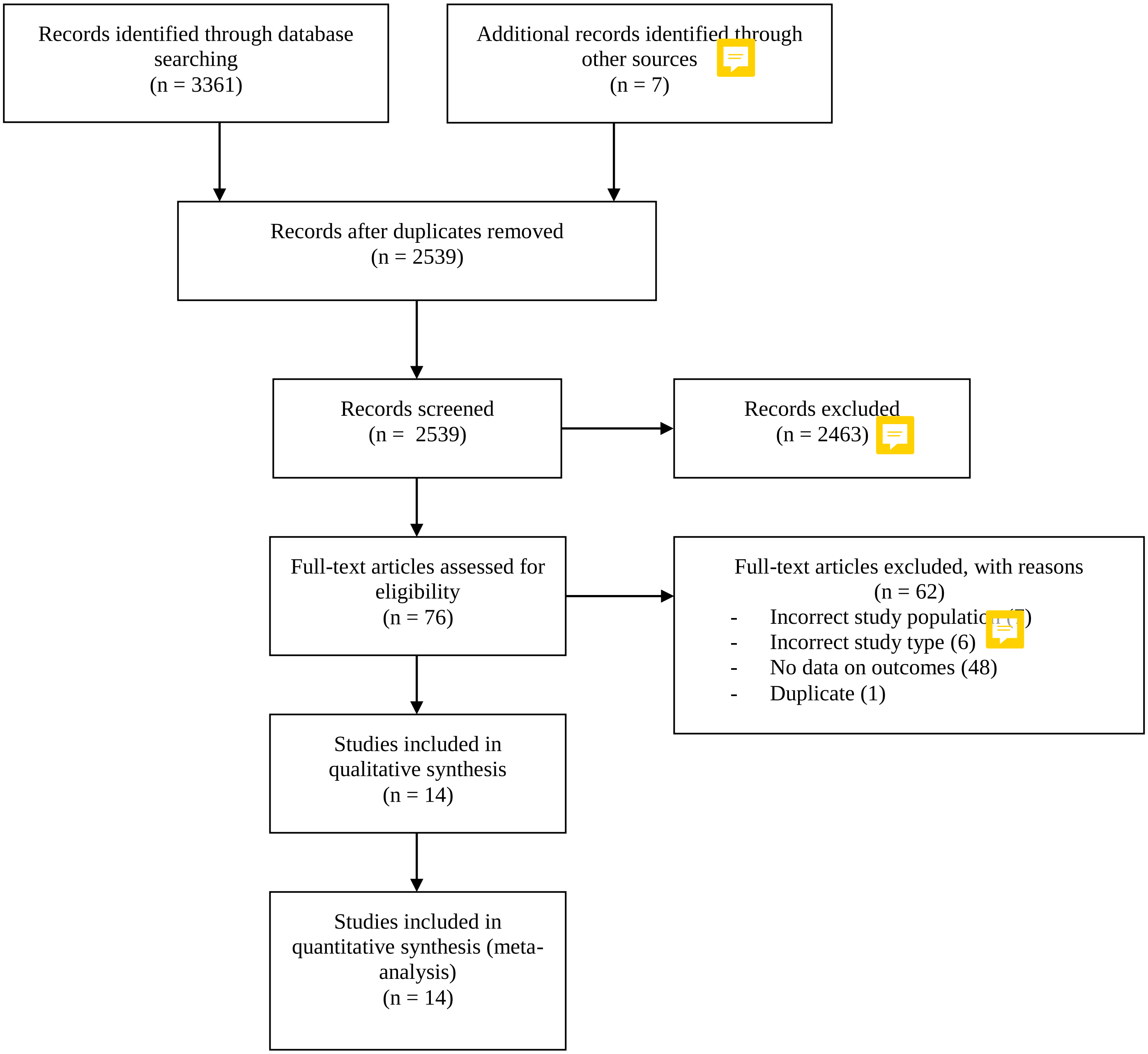

Figure

Click here to download Figure Fig. 2.pdf

Study or Subgroup	Preterm Events	Preterm Total	Term Events	Term Total	Weight	RR [95% CI]
**Intensive care unit admission**						
Assefa, 2011	6	11	0	13	1.0%	15.26 [0.96; 242.77]
Chi, 2018	102	560	142	4583	4.0%	5.88 [4.63; 7.46]
Forbes, 2010, < 33 wGA	8	40	190	1983	3.5%	2.09 [1.11; 3.94]
Forbes, 2010, 33–36 wGA	38	173	190	1983	3.9%	2.29 [1.68; 3.13]
Greenberg, 2014	13	116	15	515	3.3%	3.85 [1.88; 7.86]
Gross, 2017, < 34 wGA	12	50	19	637	3.4%	8.05 [4.15; 15.61]
Gross, 2017, 34−36 wGA	8	105	19	637	3.2%	2.55 [1.15; 5.68]
Horn, 2003, <33 wGA	11	28	60	215	3.7%	1.41 [0.85; 2.34]
Horn, 2003, 33–35 wGA	15	31	60	215	3.8%	1.73 [1.14; 2.64]
Horn, 2003, 36 wGA	9	30	60	215	3.6%	1.07 [0.60; 1.93]
Leader, 2003	20	48	12	36	3.6%	1.25 [0.71; 2.21]
Meert, 1990	16	127	12	342	3.3%	3.59 [1.75; 7.38]
Resch, 2007	9	42	27	422	3.4%	3.35 [1.69; 6.64]
Van De Steen, 2016	110	266	130	1034	4.0%	3.29 [2.65; 4.08]
Vo, 2014	2	13	7	54	2.1%	1.19 [0.28; 5.06]
**Random effect meta−analysis**		**1640**		**12884**	**49.7%**	**2.61 [1.92; 3.55]**
Heterogeneity: $Tau^2 = 0.2516$; $Chi^2 = 83.36$, df = 14 (P < 0.0001); $I^2 = 83.2\%$ [73.6%; 89.3%]						
**Emergency department visits**						
Horn, 2003, <33 wGA	22	28	145	215	4.0%	1.17 [0.94; 1.44]
Horn, 2003, 33–35 wGA	16	31	145	215	3.9%	0.77 [0.54; 1.09]
Horn, 2003, 36 wGA	23	30	145	215	4.0%	1.14 [0.91; 1.41]
**Random effect meta−analysis**		**89**		**645**	**11.9%**	**1.08 [0.94; 1.24]**
Heterogeneity: $Tau^2 = < 0.0001$; $Chi^2 = 4.34$, df = 2 (P = 0.1140); $I^2 = 54\%$ [0%; 86.8%]						
**Mechanical ventilation utilization**						
Assefa, 2011	3	11	0	13	0.9%	8.22 [0.47; 143.04]
Chi, 2018	35	560	18	4583	3.6%	15.91 [9.07; 27.91]
Followell, 2018	30	40	70	102	4.0%	1.09 [0.88; 1.36]
Gijtenbeek, 2015, < 32 wGA	1	17	3	7	1.4%	0.14 [0.02; 1.10]
Gijtenbeek, 2015, 32−36 wGA	6	38	3	7	2.6%	0.37 [0.12; 1.14]
Meert, 1990	15	127	8	342	3.1%	5.05 [2.19; 11.62]
**Random effect meta−analysis**		**793**		**5054**	**15.7%**	**1.89 [0.51; 7.06]**
Heterogeneity: $Tau^2 = 2.2227$; $Chi^2 = 95.99$, df = 5 (P < 0.0001); $I^2 = 94.8\%$ [91.1%; 96.9%]						
**Supplemental oxygen utilization**						
Gijtenbeek, 2015, < 32 wGA	14	17	6	7	3.8%	0.96 [0.66; 1.40]
Gijtenbeek, 2015, 32−36 wGA	23	38	6	7	3.8%	0.71 [0.47; 1.05]
Meert, 1990	50	127	65	342	3.9%	2.07 [1.52; 2.82]
Van De Steen, 2016	194	266	479	1034	4.1%	1.57 [1.43; 1.74]
Vo, 2014	12	13	52	54	4.0%	0.96 [0.81; 1.13]
**Random effect meta−analysis**		**461**		**1444**	**19.7%**	**1.18 [0.85; 1.64]**
Heterogeneity: $Tau^2 = 0.1187$; $Chi^2 = 46.69$, df = 4 (P < 0.0001); $I^2 = 91.4\%$ [83%; 95.7%]						
**Case fatality rate**						
Followell, 2018	0	40	0	102	0.0%	
Greenberg, 2014	0	116	1	515	0.8%	1.47 [0.06; 35.98]
Gross, 2017, < 34 wGA	0	50	0	637	0.0%	
Gross, 2017, 34−36 wGA	0	105	0	637	0.0%	
Leader, 2003	0	132	0	352	0.0%	
Van De Steen, 2016	7	266	3	1034	2.3%	9.07 [2.36; 34.84]
**Random effect meta−analysis**		**709**		**3277**	**3.0%**	**6.90 [2.00; 23.84]**
Heterogeneity: $Tau^2 = < 0.0001$; $Chi^2 = 1.05$, df = 1 (P = 0.3044); $I^2 = 5.2\%$						
**Overall random effect meta−analysis**		**3692**		**23304**	**100.0%**	**1.98 [1.45; 2.69]**
Residual heterogeneity: Tau^2 = NA; $Chi^2 = 231.45$, df = 26 (P < 0.0001); $I^2 = 88.8\%$ [84.9%; 91.7%]						

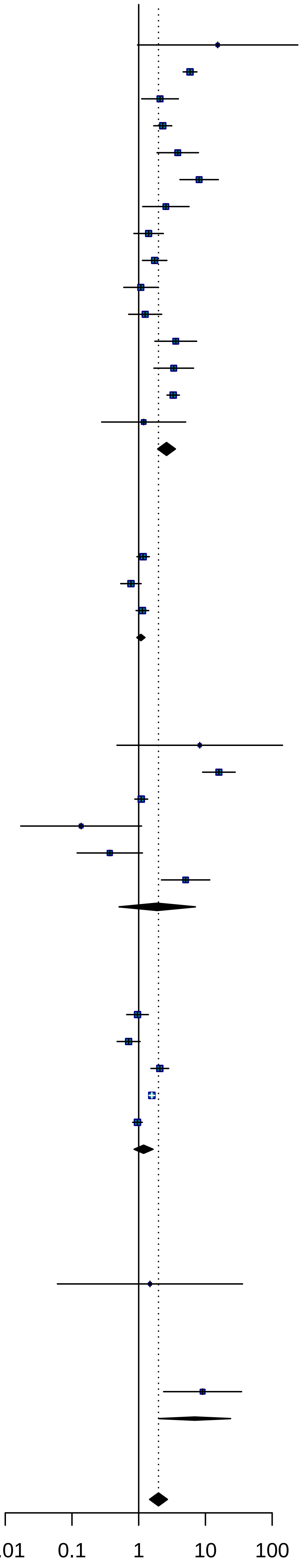

Figure

Click here to download Figure Fig. 3.pdf

Study or Subgroup	Preterm Mean	Preterm SD	Preterm Total	Term Mean	Term SD	Term Total	Weight	SMD [95% CI]
**Hospitalization LOS**								
Forbes, 2010, < 33 wGA	4.90	3.4000	40	3.40	3.4000	1983	4.2%	0.44 [0.13; 0.75]
Forbes, 2010, 33–36 wGA	5.30	5.3000	173	3.40	3.4000	1983	5.0%	0.53 [0.37; 0.69]
Gijtenbeek, 2015, < 32 wGA	8.00	2.7000	17	7.00	1.3000	7	1.7%	0.40 [−0.49; 1.29]
Gijtenbeek, 2015, 32−36 wGA	7.00	3.7000	38	7.00	1.3000	7	2.0%	0.00 [−0.81; 0.81]
Helfrich, 2015, 33–34 wGA	3.00	1.4000	164	2.00	1.4000	6954	5.0%	0.71 [0.56; 0.87]
Helfrich, 2015, 35–36 wGA	2.60	1.4000	479	2.00	1.4000	6954	5.2%	0.43 [0.34; 0.52]
Horn, 2003, <33 wGA	6.80	5.8000	28	4.10	2.8000	215	3.7%	0.82 [0.42; 1.22]
Horn, 2003, 33–35 wGA	8.40	6.9000	31	4.10	2.8000	215	3.8%	1.20 [0.81; 1.59]
Horn, 2003, 36 wGA	4.90	3.6000	30	4.10	2.8000	215	3.8%	0.27 [−0.11; 0.66]
Leader, 2003	7.00	7.5000	48	3.40	2.6000	36	3.5%	0.60 [0.16; 1.04]
Meert, 1990	5.40	5.5000	127	3.60	2.2000	342	4.8%	0.53 [0.32; 0.73]
Van De Steen, 2016	17.00	20.0000	266	8.00	5.0000	1034	5.0%	0.89 [0.75; 1.03]
**Random effect meta−analysis**			**1441**			**19945**	**47.6%**	**0.61 [0.46; 0.76]**
Heterogeneity: $Tau^2 = 0.0408$; $Chi^2 = 49.57$, df = 11 (P < 0.0001); $I^2 = 77.8\%$ [61.6%; 87.2%]								
**LOS in ICU**								
Followell, 2018	8.00	5.3000	40	6.00	3.0000	102	3.9%	0.53 [0.15; 0.90]
Forbes, 2010, < 33 wGA	5.40	3.2000	40	3.40	4.4000	1983	4.2%	0.46 [0.14; 0.77]
Forbes, 2010, 33–36 wGA	5.70	4.8000	173	3.40	4.4000	1983	5.0%	0.52 [0.36; 0.67]
Horn, 2003, <33 wGA	5.80	5.5000	28	3.80	4.1000	215	3.8%	0.47 [0.07; 0.86]
Horn, 2003, 33–35 wGA	7.70	5.5000	31	3.80	4.1000	215	3.8%	0.90 [0.52; 1.29]
Horn, 2003, 36 wGA	4.20	2.3000	30	3.80	4.1000	215	3.8%	0.10 [−0.28; 0.48]
Van De Steen, 2016	13.00	15.0000	266	6.00	5.0000	1034	5.0%	0.86 [0.72; 1.00]
**Random effect meta−analysis**			**608**			**5747**	**29.5%**	**0.57 [0.37; 0.77]**
Heterogeneity: $Tau^2 = 0.0496$; $Chi^2 = 24.02$, df = 6 (P = 0.0005); $I^2 = 75\%$ [47%; 88.2%]								
**Age at time of index HRSV infection**								
Forbes, 2010, < 33 wGA	121.00	66.0000	40	95.00	73.0000	1983	4.2%	0.36 [0.04; 0.67]
Forbes, 2010, 33–36 wGA	99.00	77.0000	173	95.00	73.0000	1983	5.0%	0.05 [−0.10; 0.21]
Helfrich, 2015, 33–34 wGA	3.90	3.6000	164	4.00	4.2000	6954	5.0%	−0.02 [−0.18; 0.13]
Helfrich, 2015, 35–36 wGA	4.20	4.8000	479	4.00	4.2000	6954	5.2%	0.05 [−0.05; 0.14]
Leader, 2003	4.00	4.0000	48	4.20	2.5000	36	3.6%	−0.06 [−0.49; 0.37]
**Random effect meta−analysis**			**904**			**17910**	**22.9%**	**0.05 [−0.03; 0.13]**
Heterogeneity: $Tau^2 = 0.0015$; $Chi^2 = 4.79$, df = 4 (P = 0.3091); $I^2 = 16.6\%$ [0%; 82.6%]								
**Overall random effect meta−analysis**			**2953**			**43602**	**100.0%**	**0.47 [0.33; 0.61]**
Residual heterogeneity: Tau^2 = NA; $Chi^2 = 78.39$, df = 21 (P < 0.0001); $I^2 = 73.2\%$ [59.1%; 82.4%]								

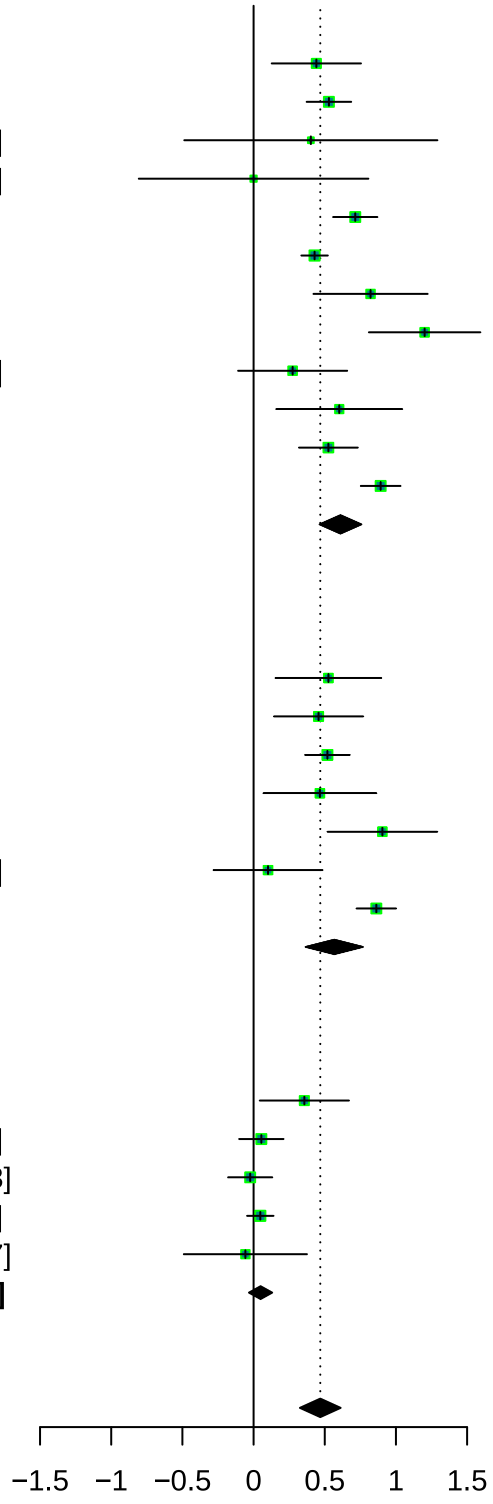

Click here to access/download
Supporting Information
Appendix_preterm vs term_PO.pdf
