# Supplementary material for: Comparison of health care resource utilization among preterm and term infants hospitalized with Human Respiratory Syncytial Virus infections: A systematic review and meta-analysis of retrospective cohort studies
Source: PLoS One. 2020 Feb 21;15(2):e0229357. doi: 10.1371/journal.pone.0229357 (PMC7034889; doi:10.1371/journal.pone.0229357)
Supplement: Attachment — Submitted filename: Response to Reviewers PONE-D-19-26852.doc [file pone.0229357.s021.doc]

**Review Comments to the Author**

Reviewer #1: Dear Authors,

I congratulate you on this very relevant piece of work.

Authors: We thank the reviewer for this appreciation.

The following are my queries/suggested modifications -

1. Were studies published in other languages apart from English also included in the analysis? (Line number 114)

Authors: We plan to translate articles published in a language other than English using Google Translate. We planned this before we search for items in the various databases. However, no eligible articles were in a language other than English. All included articles are thus published in English.

2. Was there any attempt to look for grey literature/unpublished literature? (Line number 120)

Authors: We have no plans to consider gray / unpublished literature in the work. Only articles published in peer-reviewed newspapers were considered in the review. We have now reformulated line 120 to remove any confusion.

3. This eligibility was defined as per convenience of available software. This would miss out assessment of outcome if a certain outcome is present in two published articles. This may be mentioned as a limitation? (Line number 149)

Authors: Thank you for this valuable suggestion. We added the following sentences in the discussion section: “To guarantee the robustness of our statistical analyses, we have limited our meta-analysis to outcomes available in at least 3 articles. Therefore, other relevant outcomes present in less than 3 articles including outpatient visits, antiviral or antibiotic treatment have not been included [64–66].”

4. Here it is mentioned as 75 but in Figure 1, the number is 76. Please correct. (Line number 183)

Authors: Thank you for this comment. We have revised as requested. The right number is 76.

5. Please give the number of such studies. (Line number 200)

Authors: Thank you for this comment. We have revised as requested. We added the following sentence: “Five studies [53–57], however, excluded participants with comorbidities, thereby reducing the confounding factors of these parameters on the effect of prematurity on health care utilization reported by the study.”

6. This may not be a limitation as with a thorough search, the number of articles was limited on this particular issue, so this cannot be taken as a limitation of methodology. (Line number 358)

Authors: Thank you for this comment. We have removed the limitation.

7. Stakeholders can be specified as hospital management , programme managers and policy makers. (Line number 373)

Authors: Thank you for this comment. We have revised as requested. We added the following sentence: Pediatricians and programme managers and policy makers should be aware that compared to FT children, early and late PT hospitalized for HRSV infections are at high risk for increased use of medical resources and poor outcomes.

8. One recommendation could be to take special precaution for those < 2 years of age as that is a finding of the present study

Authors: Thank you for this comment. We have revised as requested. We added the following sentence: Special attention should be paid to PT infants < 2 years.

9. This recommendation is not in line with the results obtained, so should be avoided (Line number 376)

Authors: Thank you for this comment. We have removed this recommendation.

10. In figure 1, for n=7, What are the other sources? For n=2463, a break up should be given regarding the reasons for exclusion as done for full text articles. For incorrect study type=6, does this mean incorrect study design? Then better to mention as study design.

Authors: Thank you for this comment. The other sources represent the articles we found through the screening of the list of references of the included articles and of the relevant reviews that we examined. We have now specified in figure 1 the main reasons considered for the elimination of the 2463 articles which the titles and abstracts have been screened. We have now specified the study design in Figure 1.

Reviewer #2: Dear authors

Thank you very much for the laudable effort you have put in conducting this useful piece of systematic review. I have some concerns that should be addressed as outlined below

Authors: We thank the reviewer for this appreciation.

General comments

1. Please elaborte HRSV in the abstract

Authors: Thank you for this comment. We have revised as requested. The HRSV (Human Respiratory Syncytial Virus) is now defined at the first use.

2. Please elaborate all the abbreviations when used first in the manuscript (e,g, wGA, PT, FT, ICU, ED, MV, ICD, etc)

Authors: Thank you for this comment. We have revised as requested. The lines where all the article abbreviations were defined when they were first used are specified below.

CFR (line 59)

ICU (line 56)

ED (line 56)

PT (line 68)

FT (line 68)

wGA (line 74)

LOS (line 132)

ICD (line 160)

3. When using the abbreviations, please follow the uniformity (either wGA or WGA , and not interchangeably)

Authors: Thank you for this comment. We have revised as requested.

4. I square is looking like I2

Authors: Thank you for this comment. All the values of I square presented as I2 have now been changed to I^2^
