# Supplementary material for: Comparison of health care resource utilization among preterm and term infants hospitalized with Human Respiratory Syncytial Virus infections: A systematic review and meta-analysis of retrospective cohort studies
Source: PLoS One. 2020 Feb 21;15(2):e0229357. doi: 10.1371/journal.pone.0229357 (PMC7034889; doi:10.1371/journal.pone.0229357)
Supplement: Attachment — Submitted filename: Review 2.docx [file pone.0229357.s022.docx]

Dear Authors,

Thanks for your responses. The following are my comments based on your responses -

With reference to comment made earlier under Point no. 2, since grey and unpublished literature was not searched, this should be mentioned as a limitation of the study.

With reference to comment made earlier under Point no. 10, I could not find the corrections made in Figure 1, although the authors have said that they have made corrections in Figure 1.
