# Supplementary material for: Comparison of health care resource utilization among preterm and term infants hospitalized with Human Respiratory Syncytial Virus infections: A systematic review and meta-analysis of retrospective cohort studies
Source: PLoS One. 2020 Feb 21;15(2):e0229357. doi: 10.1371/journal.pone.0229357 (PMC7034889; doi:10.1371/journal.pone.0229357)
Supplement: Attachment — Submitted filename: Response to Reviewers PONE-D-19-26852.doc [file pone.0229357.s023.doc]

**Review Comments to the Author**

Reviewer #1: Dear Authors,

Thanks for your responses. The following are my comments based on your responses -

With reference to comment made earlier under Point no. 2, since grey and unpublished literature was not searched, this should be mentioned as a limitation of the study.

Authors: Thank for this suggestion. We have added a sentence in the Discussion section accordingly: *“While Grey literature or unpublished evidence would have made an important contribution to this systematic review [65], we nevertheless covered several electronic databases including Embase which represents a database which produces unique references and we conducted robust analyses attesting that the present work is not significantly subject of publication bias [66].”*

With reference to comment made earlier under Point no. 10, I could not find the corrections made in Figure 1, although the authors have said that they have made corrections in Figure 1.

Authors: Thank you for this important reminder. Unfortunately, we provided the old version of Figure 1 after the revision. We have now provided updated Figure 1. We did not consult the full texts of the excluded articles after reading the title and summary. We cannot therefore provide details similar to that of the studies for which we have consulted the full texts, in addition this recommendation is not part of the Preferred Reporting Items for Systematic Reviews and Meta-Analyses (PRISMA) guidelines.

Reviewer #2: Dear Authors

Thank you for addressing all the comments and revising the paper accordingly

Bets wishes

We thank the reviewer for their thoughtful comments.
